# Resting-State Functional Connectivity of the Ageing Female Brain—Differences Between Young and Elderly Female Adults on Multislice Short TR rs-fMRI

**DOI:** 10.3389/fneur.2021.645974

**Published:** 2021-07-12

**Authors:** Przemysław Podgórski, Marta Waliszewska-Prosół, Anna Zimny, Marek Sąsiadek, Joanna Bladowska

**Affiliations:** ^1^Department of General and Interventional Radiology and Neuroradiology, Wroclaw Medical University, Wrocław, Poland; ^2^Department of Neurology, Wroclaw Medical University, Wrocław, Poland

**Keywords:** ageing, functional connectivity, resting-state fMRI, brain networks, SPC, NBS, FNC, elderly women

## Abstract

**Introduction:** Age-related brain changes are one of the most important world health problems due to the rising lifespan and size of the elderly populations. The aim of the study was to assess the effect of ageing in women on coordinated brain activity between eight resting-state networks.

**Material and Methods:** The study group comprised 60 healthy female volunteers who were divided into two age groups: younger women (aged 20–30 *n* = 30) and older women (aged 55–80 *n* = 30). Resting-state data were collected during a 15 min scan in the eyes-closed condition using a 3T MR scanner. Data were preprocessed and analysed using the CONN toolbox version 19.c. The large-scale network analysis included *a priori* selected regions of interest of the default mode, the sensorimotor, the visual, the salience, the dorsal attention, the fronto-parietal, the language, and the cerebellar network.

**Results:** Within the visual, the default mode, the salience, and the sensorimotor network, the intra-network resting-state functional connectivity (RSFC) was significantly higher with increasing age. There was also a significant increase in the inter-network RSFC in older females compared to young females found in the following networks: sensorimotor lateral and salience, salience and language, salience and fronto-parietal, cerebellar anterior and default mode, cerebellar posterior and default mode, visual and sensorimotor lateral, visual and sensorimotor, visual lateral and default mode, language and cerebellar anterior, language and cerebellar posterior, fronto-parietal and cerebellar anterior, dorsal attention and sensorimotor, dorsal attention and default mode, sensorimotor superior, and salience. Compared to young females, elderly women presented bilaterally significantly lower inter-network RSFC of the salience supramarginal gyrus and cerebellar posterior, sensorimotor lateral, and cerebellar anterior network, and sensorimotor lateral and cerebellar posterior as well as sensorimotor superior and cerebellar posterior network.

**Conclusion:** Increased RSFC between some brain networks including the visual, the default mode, the salience, the sensorimotor, the language, the fronto-parietal, the dorsal attention, and the cerebellar networks in elderly females may function as a compensation mechanism during the ageing process of the brain. To the best of our knowledge, this study is the first to report the importance of increase of cerebellar networks RSFC during healthy female ageing.

## Introduction

Ageing is one of the most important problems of contemporary society due to the increasing lifespan and size of elderly populations. There are known differences in the ageing process between women and men. Currently and historically, women tend to live longer than men worldwide according to reliable demographic data. On the other hand, women have a higher prevalence of chronic, age-related degenerative diseases than men (1). Sex differences influence brain morphology and physiology during both development and ageing. Men have larger brains in comparison with women (2, 3). At the same time, metabolic brain activity is significantly lower in females (4, 5). Furthermore, neuroanatomical sex differences in the brain are region-specific (2, 3). Apart from the anatomical dissimilarities, recently there have also been reports on differences associated with sex in resting-state functional connectivity (RSFC) (6, 7).

On the other hand, men more often turn to unhealthy behaviours such as smoking, use of psychoactive substances, poor diet, and alcohol consumption which contribute to the increased risk of cardiovascular, and other chronic diseases and higher mortality in men (8). In order to exclude the influence of cerebrovascular disease on resting-state RSFC changes, female subjects presenting a normal-appearing brain in conventional magnetic resonance imaging (MRI) seem to be suitable candidates to be included in a study group appropriate for the analysis of the normal brain ageing process.

Therefore, this paper explores the intra- and inter-network functional connectivity (FC) differences between the young female and the older female brain based on resting-state functional MRI (rs-fMRI) in order to avoid any impact of sex on age-related RSFC changes.

Resting-state fMRI enables the assessment of synchronous activations between brain areas that are spatially separate which occur in the absence of a stimulus or task. This method is non-invasive and does not require patient cooperation in order to identify resting-state networks in the brain (9, 10).

In general, in fMRI studies investigating resting-state networks, greater attention has been paid to the default mode network (DMN). The DMN contains a range of different cortical areas constantly found to be working at rest, including the medial prefrontal cortex, the inferior parietal lobule, the hippocampus, and the posterior cingulate cortex/retrosplenial cortex/precuneus (a group of regions also called the posteromedial cortex) (10). Moreover, the DMN plays a very important role in research studies concerning the ageing process, mainly due to special areas included in this network, such as the posterior cingulate cortex and the hippocampus, both of which are involved fundamentally in the pathogenesis of Alzheimer's disease (10, 11).

Obviously, the DMN is the most frequently investigated large-scale resting-state network in the studies of age-related RSFC changes; however, the results are often inconclusive indicating that further investigations are needed (10, 12–14). Moreover, many reports on brain ageing have demonstrated decreased RSFC not only in the DMN but also within other networks, such as the salience network and the motor network (10, 12, 15–18).

The aim of the study was to investigate the positive and negative effects of ageing in female adults on the coordinated activity between large-scale resting-state networks (RSNs) that are important for high-level cognitive functions. The purpose of our research was also to assess the influence of healthy ageing on RSFC network connectivity in women as well as trying to find out which network could act as a possible compensatory mechanism.

We performed an ROI-to-ROI analysis focusing on eight RSNs, including the DMN, the salience network (SAL), the fronto-parietal network (FPN), the dorsal attention network (DAN), the sensorimotor network (SMN), the language network (LAN), the visual network (VIS), and the cerebellar (CER) network.

## Material

Sixty healthy female volunteers without neurological and cognitive deficits were enrolled in the study. The subjects were divided into two age groups: group 1—younger adults (aged 20–30 years; mean age 23.9 years; *n* = 30) and group 2—older adults (aged 55–80; mean age 63.3 years; *n* = 30). The main radiological inclusion criterium was a normal-appearing brain on conventional MRI, with up to only five white matter hyperintensities.

Healthy right-handed female volunteers without past or present neurological, and cognitive deficits with at least +12 years of education and native Polish language skills. All participants had to be able to perform all activities of daily living independently.

All subjects in the study group had higher education and worked in their professions. Forty-five women (75%) live in a large city (over 500,000 inhabitants), and the remaining 15 women (25%) live in smaller towns and villages.

Additional demographic data are shown in Table 1.

**Table 1:** Demographic and clinical data for both groups.

			**Statistics *t*-test**
**Demographic data**	**Young women**	**Elderly women**	***p* value**
Age	23.9 ± 2.9	63.3 ± 8.5	<0.0001
Education (years)	13.6 ± 1.7	14 ± 1.9	0.409
EHI score	78.5 ± 21.2	74.5 ± 24.5	0.508
MMSE	29.2 ± 0.67	28.8 ± 0.76	0.056
CDT	10	10	–

*EHI, Edinburgh Handedness Inventory; MMSE, Mini-Mental State Examination; CDT, Clock-drawing test*.

The exclusion criteria were as follows:

1. Presence of any neurological disease (e.g., cerebrovascular disease, post-toxic changes, inflammatory changes, multiple sclerosis, brain neoplasms);
2. Any autoimmune diseases, endocrinopathy and metabolic diseases (e.g., diabetes, cardiovascular diseases, systemic lupus erythematosus, Hashimoto's thyroiditis, Sjögren's syndrome);
3. A history of psychiatric disease (e.g., depression, schizophrenia, ADHD, autism);
4. Chronic use of CNS-active medications (e.g., neuroleptics, antiepileptic drugs, psychostimulants, steroids, analgesics, sedatives);
5. Active alcohol or drug abuse;
6. Visual and hearing impairment and/or symptoms of a neurological or cognitive deficit;
7. Any structural brain abnormality presented on conventional MR imaging;
8. Current pregnancy and breastfeeding, hormonal contraceptives;
9. A history of head trauma with loss of consciousness;
10. Being underweight (BMI < 18.5 kg/m^2^) or obese with BMI ≥ 35 kg/m^2^;
11. Claustrophobia.

## Methods

### Participants

A prospective observational study was supported by the Wroclaw Medical University Grant SUB.C270.21.020 and conducted in accordance with the guidelines of the Wroclaw Medical University Ethics Committee for conducting research involving humans permission no. KB-57/2021. Each participant signed her informed consent before inclusion.

### Neurological Assessment

The neurological study protocol included a detailed neurological examination, with assessment of mental state using the Mini-Mental State Examination (MMSE), and the clock-drawing test (CDT) to screen for cognitive impairment.

### Data Acquisition

Resting-state data were collected during a 15-min scan in a 3T MR scanner (Ingenia Philips, Best, Netherlands) equipped with 45-mT/m 200-T/m/s gradients and a 32-channel head coil with foam padding to minimise head motion and noise-cancelling headphones. The study included the acquisition of a high-resolution sagittal T1-weighted sequence (number of slices = 257; repetition time (TR) = 11 ms; echo time (TE) = 5 ms; flip angle = 8°; field of view (FOV) = 256 × 256 mm; and voxel size = 0.75 × 0.75 × 0.75 mm). EPI multiband sequence: MB = 6, TR/TE = 1,100/31 ms, resolution 2.5 × 2.5 × 2.5 mm^3^ for rs-fMRI was performed. Participants were asked to stay awake during the examination with their eyes closed through the entire acquisition. After the rs-fMRI session, a verbal confirmation was used to cheque if they were awake during the rs-fMRI scanning procedure.

### Data Preprocessing

Functional and structural images were analysed using CONN, a Matlab/SPM-based software. Data were preprocessed using a standard pipeline in Statistical Parametric Mapping software (SPM12, Wellcome Department of Cognitive Neurology, University College London) running under Matlab Release 2019b (The MathWorks, Inc., Natick, MA, United States) and Linux HPC Server running on Ubuntu 18.04. The preprocessing methods applied included slice timing correction, realignment, segmentation, normalisation to the MNI template, and smoothing. Functional data were realigned using SPM12 (19) and realign and unwarp procedure (20). All scans were co-registered and resampled to a reference image using b-spline interpolation. Temporal misalignment between different slices of the functional data was corrected using the SPM12 slice-timing correction procedure (21). Identification of outlier scans was performed using the Artefact Detection Tools (ART) toolbox (https://www.nitrc.org/projects/artifact_detect/). Outlier scans were identified from the observed global BOLD signal and the amount of subject motion in the scanner. Outlier scans that exceeded three standard deviations from the global mean BOLD signal, or with framewise displacement >0.5 mm, were identified. In the next step, functional and anatomical scans were normalised into standard MNI space with a final isotropic voxel size of 1 mm and segmented into grey matter, white matter, and cerebrospinal fluid tissue classes using SPM12 unified segmentation and normalisation procedure (22). The direct normalisation procedure applied unified segmentation and normalisation procedures separately to the functional data, using the mean BOLD signal as a reference image, and to the structural data, using the raw T1-weighted volume as a reference image. Both functional and anatomical data were resampled to a default 180 × 216 × 180-mm bounding box, with 2 mm isotropic voxels for functional data and 1 mm for anatomical data, using fourth-order spline interpolation. In order to minimise the impact of motion and physiological noise factors, the CompCor (23) function was used to define and remove confounds in the blood-oxygen-level-dependent signal. In order to focus on slow-frequency fluctuations, fMRI data were bandpass filtered at 0.008–0.09 Hz using a discrete cosine transform to minimise border effects (24).

Signal contributions from cerebrospinal fluid, brain white matter, and micro-movements of the head (three translation and three rotation parameters plus their associated first-order derivatives) were identified and removed by multiple regression. In order to increase the BOLD signal-to-noise ratio and reduce the influence of residual variability in functional and gyral anatomy across subjects, functional data were smoothed using spatial convolution with a Gaussian kernel of 8-mm full width at half maximum (FWHM).

### First-Level Analysis

**Figure 1:**
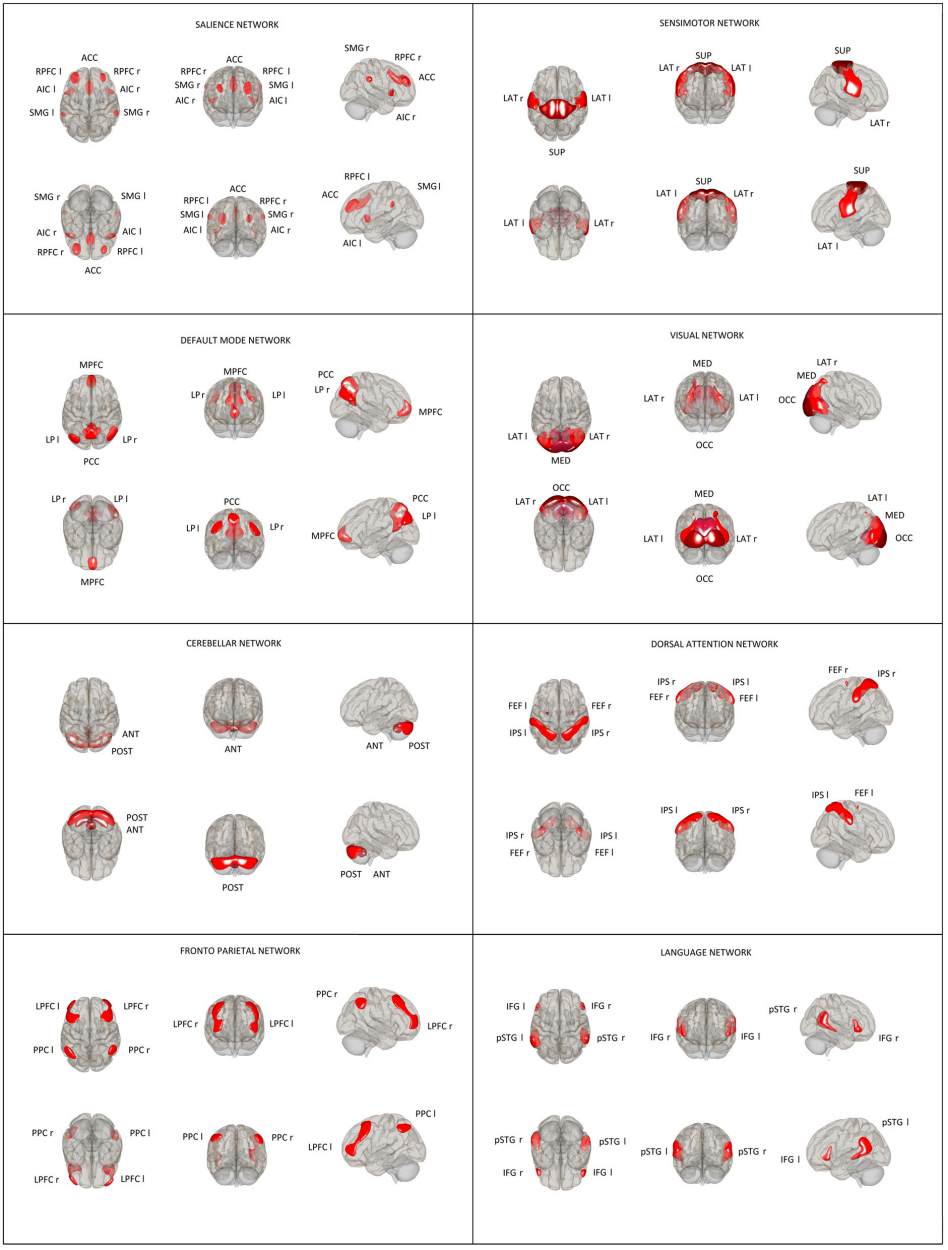
Location and size of predefined rs-network ROIs used in the ROI–ROI analysis. Default mode network (DMN): medial prefrontal cortex (MPFC), precuneus cortex (PCC), bilateral lateral parietal (LP); sensorimotor network (SMN): superior, bilateral lateral; visual network (VIS): medial, occipital, bilateral lateral; salience network (SN): anterior cingulate cortex (ACC), bilateral anterior insula (AI), rostral prefrontal cortex (RPFC), and supramarginal gyrus (SMG); dorsal attention network (DA): bilateral frontal eye field (FEF) and intraparietal sulcus (IPS); fronto-parietal network (FPN): bilateral lateral prefrontal cortex (LPFC) and posterior parietal cortex (PPC); language network (LAN): bilateral inferior frontal gyrus (IFG) and posterior superior temporal gyrus (pSTG); and cerebellar network (CER): anterior, posterior.

Thirty-two ROI seeds, with predefined shape and locations derived from the HCP atlas (25) adjusted to each volume, were used to assess eight RSNs including DMN, SAL, FPN, DAN, SMN, LAN, VIS, and CER (Figure 1). Functional connectivity measures were computed between earlier predefined seed regions of rs-networks to identify patterns of ROI-to-ROI connectivity by computing bivariate Pearson's correlation measures between the extracted mean BOLD signal time courses of each pair of ROIs. The resulting coefficients were converted to normally distributed scores using Fisher's transformation to improve normality assumptions of the subsequent, second-level analyses.

### Second-Level Analysis

To allow between-group comparisons, individual matrices were entered into a second-level general linear model. We compared FC between group 1 and group 2 scans using two-tailed paired *t*-tests. The results were reported only when surviving a false discovery rate (FDR)-corrected threshold of *p* < 0.05 at the seed level.

To address the issue of variability of the results of FC analyses which are partially dependent on the selected method used to control family-wise errors and node characteristics (26), we performed three separate analyses described in the statistics section comprising well-established methods such as functional network connectivity (FNC), spatial pairwise clustering (SPC), and network-based statistics (NBS).

### Statistical Analysis

Between-subjects [Young Adults (−1) vs. Old Adults (1)], contrasts were applied for the ROI analysis. All network nodes were used as both sources and targets for the ROI analysis. Our findings are based on SPC analysis with the parameters described below. Additionally, we present alternative results by applying various approaches to node clustering and thresholding, such as FNC and NBS.

### FNC

FNC is one of the default second-level analysis methods for the CONN toolbox that is based on an approach first described by Jafri et al. (27) used for determining the temporal dependency among the components with very weak relationships which cannot be considered as one component in the independent component analysis (ICA). FNC analyses the entire set of connexions between all pairs of ROIs in terms of intra- and inter-network connectivity sets (27). A multivariate parametric general linear model analysis for all connexions included in each of these sets/clusters of connexions was performed. As a final step for the obtained F-statistic for each pair of networks, a *p* < 0.05 was used together with an associated uncorrected cluster-level height and an FDR-corrected cluster-level threshold of *p* < 0.05 (28) to control FNC family-wise error rates.

### SPC

The SPC approach is based on a work by Zalesky et al. (29). It starts with the entire ROI-to-ROI matrix of T- or F-statistics estimated using a general linear model, forming a two-dimensional statistical parametric map which is then thresholded using an *a priori* “height” threshold. In the next step, resulting non-overlapping clusters are characterised by mass which is compared to a distribution of expected cluster mass values under the null hypothesis (25). The results are summarised, for each individual cluster or group of connexions, by uncorrected cluster-level *p*-values, cluster-level FWE-corrected *p*-values, and cluster-level FDR-corrected *p*-values. In our analyses, the following SPC parameters were used: cluster threshold: *p* < 0.05 cluster-level p-FDR corrected (SPC mass/intensity); connexion threshold: *p* < 0.01 *p* uncorrected.

### NBS

NBS is a method similar to SPC that allows making inferences about entire networks of ROIs. The clusters in NBS are defined by a graph theoretical concept of connected components (30) instead of pairwise clustering like in SPC. NBS analysis starts with the entire ROI-to-ROI matrix of T- or F-statistics estimated using a general linear model, forming a two-dimensional statistical parametric map which is then thresholded using an *a priori* “height” threshold in which order of ROIs is not relevant. Those resulting suprathreshold connexions define the graph among all nodes. In the next step, the obtained graph is broken into components/networks which are defined as connected subgraphs (25). Then, each network is characterised by its network mass but using networks instead of clusters as in SPC (25). Results are summarised, for each individual network or group of connexions, by uncorrected network-level *p*-values, network-level FWE-corrected *p*-values, and network-level FDR-corrected *p*-values.

The values used as thresholds in our study were as follows: network threshold: *p* < 0.05 network-level p-FDR corrected (NBS mass/intensity) connexion threshold: *p* < 0.001 *p*-uncorrected.

## Results

### Neurological Examination and Cognitive Function

The neurological examination and the results of the MMSE and CDT tests were within normal limits in all subjects (Table 1).

### Intra- and Inter-connectivity Pattern Analysis of Large-Scale Networks

The ROI–ROI FC analysis using the SPC method revealed a significant increase in the intra- and inter-network brain FC within 11 clusters (Table 2) and a significant decrease in the inter-network brain FC in two clusters (Table 3) in group 2 (elderly females) in comparison to group 1 (younger females).

**Table 2:** Statistical cluster analysis of the networks using spatial pairwise clustering (SPC) presenting increased connectivity between nodes.

**Analysis**		**Statistic**	***p*-unc**	***p*-FDR**	***p*-FWE**
**Cluster 1**		**Score = 110.3**	**0**	**0**	**0**
		**Mass = 398**	**0**	**0**	**0**
		**Size = 24**	**0.000043**	**0.000707**	**0**
Cerebellar.Posterior	DefaultMode.LP l	T(58) = 5.23	–	0.000002	0.00024
Cerebellar.Anterior	DefaultMode.LP r	T(58) = 5.13	–	0.000004	0.00027
Cerebellar.Anterior	DefaultMode.LP l	T(58) = 5.07	–	0.000004	0.00027
Cerebellar.Anterior	DefaultMode.PCC	T(58) = 4.61	–	0.000023	0.00063
Cerebellar.Posterior	DefaultMode.LP r	T(58) = 4.34	–	0.000058	0.00114
DefaultMode.MPFC	DefaultMode.LP l	T(58) = 4.2	–	0.000095	0.00148
DefaultMode.MPFC	DefaultMode.LP r	T(58) = 3.72	–	0.000451	0.00476
Cerebellar.Anterior	DefaultMode.MPFC	T(58) = 3.42	–	0.001157	0.0082
DefaultMode.PCC	DefaultMode.LP r	T(58) = 3.38	–	0.001319	0.00908
Cerebellar.Posterior	DefaultMode.PCC	T(58) = 3.15	–	0.002604	0.01441
Cerebellar.Posterior	DefaultMode.MPFC	T(58) = 2.83	–	0.006472	0.02925
DefaultMode.MPFC	DefaultMode.PCC	T(58) = 2.68	–	0.009627	0.03882
**Cluster 2**		**Score = 106**	**0**	**0**	**0**
		**Mass = 507.9**	**0**	**0**	**0**
		**Size = 36**	**0**	**0**	**0**
Salience.SMG r	Salience.ACC	T(58) = 5.07	–	0.000004	0.00027
Salience.Ainsula r	Salience.ACC	T(58) = 4.93	–	0.000007	0.00036
Salience.SMG l	Salience.ACC	T(58) = 4.36	–	0.000054	0.00112
Salience.Ainsula r	Salience.RPFC r	T(58) = 4.2	–	0.000094	0.00148
Salience.Ainsula r	Salience.RPFC l	T(58) = 4.07	–	0.000144	0.00204
SensoriMotor.Lateral r	Salience.RPFC r	T(58) = 3.75	–	0.000413	0.00466
SensoriMotor.Lateral r	Salience.ACC	T(58) = 3.73	–	0.000431	0.00475
SensoriMotor.Superior	Salience.SMG r	T(58) = 3.68	–	0.000507	0.00502
SensoriMotor.Lateral r	Salience.RPFC l	T(58) = 3.51	–	0.000876	0.00729
Salience.Ainsula l	Salience.RPFC l	T(58) = 3.49	–	0.000938	0.00729
Salience.SMG l	Salience.RPFC r	T(58) = 3.48	–	0.000965	0.00737
SensoriMotor.Superior	Salience.Ainsula r	T(58) = 3.46	–	0.001035	0.00766
SensoriMotor.Superior	Salience.Ainsula l	T(58) = 3.43	–	0.001128	0.0082
SensoriMotor.Lateral l	Salience.ACC	T(58) = 3.36	–	0.00139	0.00919
SensoriMotor.Lateral r	Salience.SMG r	T(58) = 3.3	–	0.001674	0.01047
SensoriMotor.Superior	Salience.ACC	T(58) = 3.16	–	0.002471	0.01401
Salience.Ainsula l	Salience.ACC	T(58) = 3.02	–	0.003759	0.01942
SensoriMotor.Lateral l	Salience.RPFC l	T(58) = 2.77	–	0.007616	0.03229
**Cluster 3**		**Score = 97.49**	**0**	**0**	**0**
		**Mass = 302.6**	**0.000064**	**0.000701**	**0**
		**Size = 18**	**0.000298**	**0.001669**	**0.002**
Salience.ACC	Language.pSTG r	T(58) = 5.32	–	0.000002	0.00022
Salience.ACC	Language.pSTG l	T(58) = 4.75	–	0.000014	0.00053
Salience.RPFC r	Language.pSTG r	T(58) = 4.7	–	0.000016	0.00054
Salience.RPFC l	Language.pSTG l	T(58) = 4	–	0.00018	0.00242
Salience.Ainsula l	Language.pSTG l	T(58) = 3.92	–	0.000236	0.00304
Salience.Ainsula r	Language.pSTG r	T(58) = 3.88	–	0.000272	0.0033
Salience.RPFC l	Language.pSTG r	T(58) = 3.52	–	0.00086	0.00729
Salience.Ainsula l	Language.pSTG r	T(58) = 3.47	–	0.000998	0.0075
Salience.RPFC r	Language.pSTG l	T(58) = 2.76	–	0.0077	0.03237
**Cluster 4**		**Score = 84.86**	**0**	**0**	**0**
		**Mass = 275.9**	**0.000107**	**0.000882**	**0.001**
		**Size = 18**	**0.000298**	**0.001669**	**0.002**
Language.IFG l	DefaultMode.PCC	T(58) = 4.73	–	0.000015	0.00053
Language.pSTG l	Cerebellar.Anterior	T(58) = 4.62	–	0.000022	0.00063
Language.IFG l	Cerebellar.Posterior	T(58) = 4.41	–	0.000045	0.00097
Language.IFG l	Cerebellar.Anterior	T(58) = 4.03	–	0.000164	0.00226
Language.pSTG l	Cerebellar.Posterior	T(58) = 3.73	–	0.000441	0.00476
Language.IFG r	Cerebellar.Anterior	T(58) = 3.67	–	0.000526	0.00502
FrontoParietal.LPFC l	DefaultMode.MPFC	T(58) = 3.41	–	0.001198	0.00837
Language.pSTG r	Cerebellar.Anterior	T(58) = 3.37	–	0.001355	0.00908
Language.IFG l	DefaultMode.MPFC	T(58) = 2.86	–	0.005848	0.02686
**Cluster 5**		**Score = 83.49**	**0**	**0**	**0**
		**Mass = 252.3**	**0.000145**	**0.000952**	**0.001**
		**Size = 16**	**0.000354**	**0.001669**	**0.003**
Visual.Medial	DorsalAttention.IPS r	T(58) = 4.82	–	0.000011	0.00045
Visual.Lateral r	Visual.Medial	T(58) = 4.64	–	0.00002	0.00062
Visual.Lateral l	Visual.Medial	T(58) = 4.41	–	0.000045	0.00097
Visual.Medial l	DorsalAttention.IPS l	T(58) = 4.11	–	0.000127	0.00185
Visual.Occipital r	DorsalAttention.IPS r	T(58) = 3.92	–	0.000239	0.00304
Visual.Occipital l	DorsalAttention.IPS l	T(58) = 3.49	–	0.000916	0.00729
Visual.Medial	Visual.Occipital	T(58) = 3	–	0.004017	0.02012
Visual.Lateral r	Visual.Occipital	T(58) = 2.92	–	0.004989	0.02335
**Cluster 6**		**Score = 59.29**	**0.00102**	**0.003538**	**0.007**
		**Mass = 217.2**	**0.000202**	**0.000952**	**0.001**
		**Size = 18**	**0.000298**	**0.001669**	**0.002**
Visual.Medial	SensoriMotor.Lateral r	T(58) = 4.57	–	0.000026	0.00068
Visual.Medial	SensoriMotor.Lateral l	T(58) = 4.22	–	0.000086	0.00148
Visual.Lateral r	SensoriMotor.Lateral l	T(58) = 3.49	–	0.000941	0.00729
Visual.Medial	SensoriMotor.Superior	T(58) = 3.37	–	0.00134	0.00908
Visual.Lateral l	SensoriMotor.Lateral l	T(58) = 3.23	–	0.002058	0.01215
Visual.Lateral r	SensoriMotor.Superior	T(58) = 3.17	–	0.002415	0.01401
Visual.Lateral l	SensoriMotor.Superior	T(58) = 3.1	–	0.002995	0.01615
Visual.Medial	DorsalAttention.FEF l	T(58) = 2.97	–	0.004273	0.02099
Visual.Lateral l	SensoriMotor.Lateral r	T(58) = 2.73	–	0.008275	0.03417
**Cluster 7**		**Score = 110.2**	**0**	**0**	**0**
		**Mass = 120.5**	**0.002404**	**0.008816**	**0.016**
		**Size = 4**	**0.185644**	**0.306312**	**0.472**
SensoriMotor.Superior	SensoriMotor.Lateral l	T(58) = 5.59	–	0.000001	0.00022
SensoriMotor.Superior	SensoriMotor.Lateral r	T(58) = 5.39	–	0.000001	0.00022
**Cluster 8**		**Score = 38.33**	**0.006119**	**0.015532**	**0.038**
		**Mass = 109.8**	**0.002895**	**0.009554**	**0.018**
		**Size = 10**	**0.006029**	**0.022106**	**0.033**
DorsalAttention.IPS l	SensoriMotor.Superior	T(58) = 3.85	–	0.000293	0.00346
DorsalAttention.IPS l	SensoriMotor.Lateral l	T(58) = 3.42	–	0.001153	0.0082
DorsalAttention.IPS r	SensoriMotor.Lateral l	T(58) = 3.3	–	0.001655	0.01047
DorsalAttention.IPS r	SensoriMotor.Superior	T(58) = 2.98	–	0.004248	0.02099
DorsalAttention.IPS l	SensoriMotor.Lateral r	T(58) = 2.93	–	0.004861	0.02296
**Cluster 9**		**Score = 57.76**	**0.001072**	**0.003538**	**0.007**
		**Mass = 78.32**	**0.010725**	**0.029751**	**0.051**
		**Size = 4**	**0.185644**	**0.306312**	**0.472**
FrontoParietal.PPC l	Cerebellar.Anterior	T(58) = 5.49	–	0.000001	0.00022
FrontoParietal.PPC r	Cerebellar.Anterior	T(58) = 3	–	0.004004	0.02012
**Cluster 10**		**Score = 28.72**	**0.027642**	**0.057012**	**0.113**
		**Mass = 78.02**	**0.010818**	**0.029751**	**0.051**
		**Size = 8**	**0.013605**	**0.044896**	**0.064**
DorsalAttention.IPS r	Salience.RPFC r	T(58) = 3.5	–	0.000891	0.00729
DorsalAttention.FEF r	Salience.RPFC r	T(58) = 3.35	–	0.001412	0.00922
DorsalAttention.FEF r	Salience.ACC	T(58) = 2.88	–	0.005612	0.02602
DorsalAttention.FEF l	Salience.RPFC l	T(58) = 2.69	–	0.00941	0.03826
**Cluster 11**		**Score = 50.87**	**0.001557**	**0.00467**	**0.01**
		**Mass = 71.96**	**0.013226**	**0.033574**	**0.062**
		**Size = 4**	**0.185644**	**0.306312**	**0.472**
Visual.Lateral r	DefaultMode.PCC	T(58) = 4.28	–	0.000071	0.0013
Visual.Lateral l	DefaultMode.PCC	T(58) = 4.2	–	0.000093	0.00148

*p-unc, uncorrected p-value; p-FDR, false discovery rate-adjusted p-value*.

*p-FWE, family-wise error rate-adjusted p-value*.

*DMN, Default mode network; MPFC, medial prefrontal cortex; PCC, precuneus cortex; LP, bilateral lateral parietal cortex; SMN, sensorimotor network; VIS, superior, bilateral lateral; visual network; medial, occipital, bilateral lateral; SN, salience network; ACC, anterior cingulate cortex; AI, bilateral anterior insula; RPFC, rostral prefrontal cortex; SMG, supramarginal gyrus; DA, dorsal attention network; FEF, bilateral frontal eye field; IPS, intraparietal sulcus; FPN, fronto-parietal network; LPFC, bilateral lateral prefrontal cortex; PPC, posterior parietal cortex; LAN, language network; IFG, bilateral inferior frontal gyrus; pSTG, posterior superior temporal gyrus; CER, cerebellar network: anterior, posterior*.

*The bold values are cluster statistic*.

**Table 3:** Statistical cluster analysis of the networks using spatial pairwise clustering (SPC) showing decreased connectivity between nodes.

			***p*-unc**	***p*-FDR**	***p*-FWE**
**Cluster 1**		**70.38**	**0.000474**	**0.002233**	**0.002**
		**Mass = 225.1**	**0.000189**	**0.000952**	**0.001**
		**Size = 16**	**0.000354**	**0.001669**	**0.003**
Salience.SMG r	Cerebellar.Posterior	T(58) = −4.95	–	0.000007	0.00036
SensoriMotor.Lateral r	Cerebellar.Anterior	T(58) = −4.14	–	0.000113	0.00169
Salience.Ainsula r	Cerebellar.Posterior	T(58) = −3.69	–	0.000496	0.00502
SensoriMotor.Lateral l	Cerebellar.Anterior	T(58) = −3.58	–	0.000704	0.00646
Salience.SMG l	Cerebellar.Posterior	T(58) = −3.54	–	0.000803	0.00699
SensoriMotor.Lateral l	Cerebellar.Posterior	T(58) = −3.31	–	0.001629	0.01047
SensoriMotor.Superior	Cerebellar.Posterior	T(58) = −3.26	–	0.001873	0.01147
SensoriMotor.Lateral r	Cerebellar.Posterior	T(58) = −3.23	–	0.00205	0.01215
**Cluster 2**		**Score = 62.79**	**0.000691**	**0.002849**	**0.004**
		**Mass = 190.9**	**0.000253**	**0.001042**	**0.002**
		**Size = 14**	**0.001123**	**0.004634**	**0.008**
Salience.SMG r	FrontoParietal.LPFC l	T(58) = −4.9	–	0.000008	0.00036
Salience.SMG r	Language.IFG l	T(58) = −4.33	–	0.00006	0.00114
Salience.SMG l	Language.IFG l	T(58) = −3.68	–	0.000518	0.00502
Salience.SMG l	FrontoParietal.LPFC l	T(58) = −3.66	–	0.000554	0.00519
Salience.Ainsula r	FrontoParietal.LPFC l	T(58) = −3.06	–	0.003366	0.01772
Salience.SMG r	Language.pSTG l	T(58) = −3	–	0.004016	0.02012
Salience.Ainsula r	Language.IFG l	T(58) = −2.73	–	0.008322	0.03417

*p-unc, uncorrected p-value; p-FDR, false discovery rate-adjusted p-value; p-FEW, family-wise error rate-adjusted p-value*.

*DMN, Default mode network; MPFC, medial prefrontal cortex; PCC, precuneus cortex; LP, bilateral lateral parietal cortex; SMN, sensorimotor network; VIS, superior, bilateral lateral; visual network; medial, occipital, bilateral lateral; SN, salience network; ACC, anterior cingulate cortex; AI, bilateral anterior insula; RPFC, rostral prefrontal cortex; SMG, supramarginal gyrus; DA, dorsal attention network; FEF, bilateral frontal eye field; IPS, intraparietal sulcus; FPN, fronto-parietal network; LPFC, bilateral lateral prefrontal cortex; PPC, posterior parietal cortex; LAN, language network; IFG, bilateral inferior frontal gyrus; pSTG, posterior superior temporal gyrus; CER, cerebellar network: anterior, posterior*.

*The bold values are cluster statistic*.

**Figure 2:**
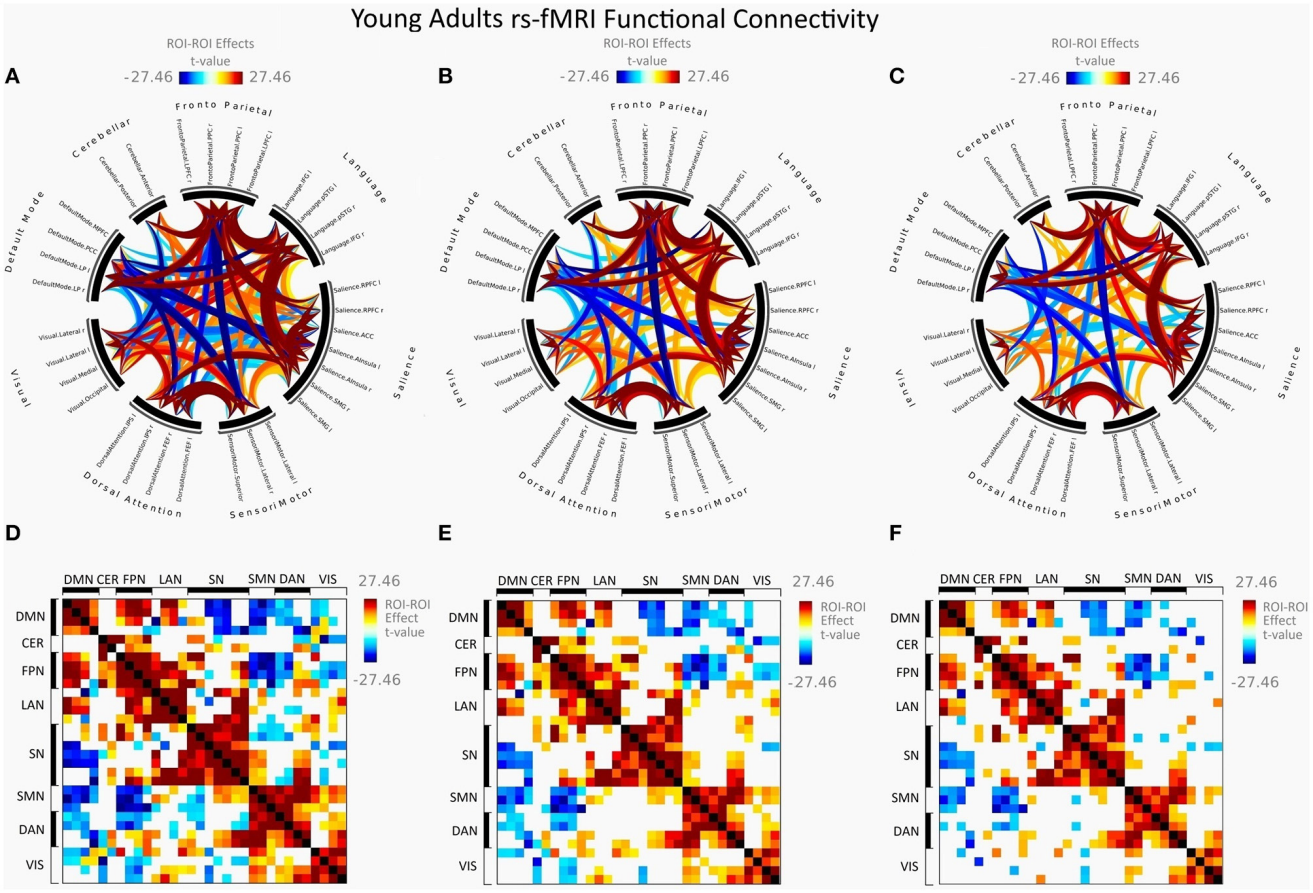
Mean FC in female young adults presented as a connectome ring and a connectivity matrix showing nodes with increased FC (red) and decreased FC (blue). Connectome ring network correlations computed using: **(A)** Functional network connectivity (FNC). **(B)** Spatial pairwise clustering (SPC). **(C)** Network-based statistics (NBS). Connectome matrix network correlations computed using: **(D)** Functional network connectivity (FNC). **(E)** Spatial pairwise clustering (SPC). **(F)** Network-based statistics (NBS).

**Figure 3:**
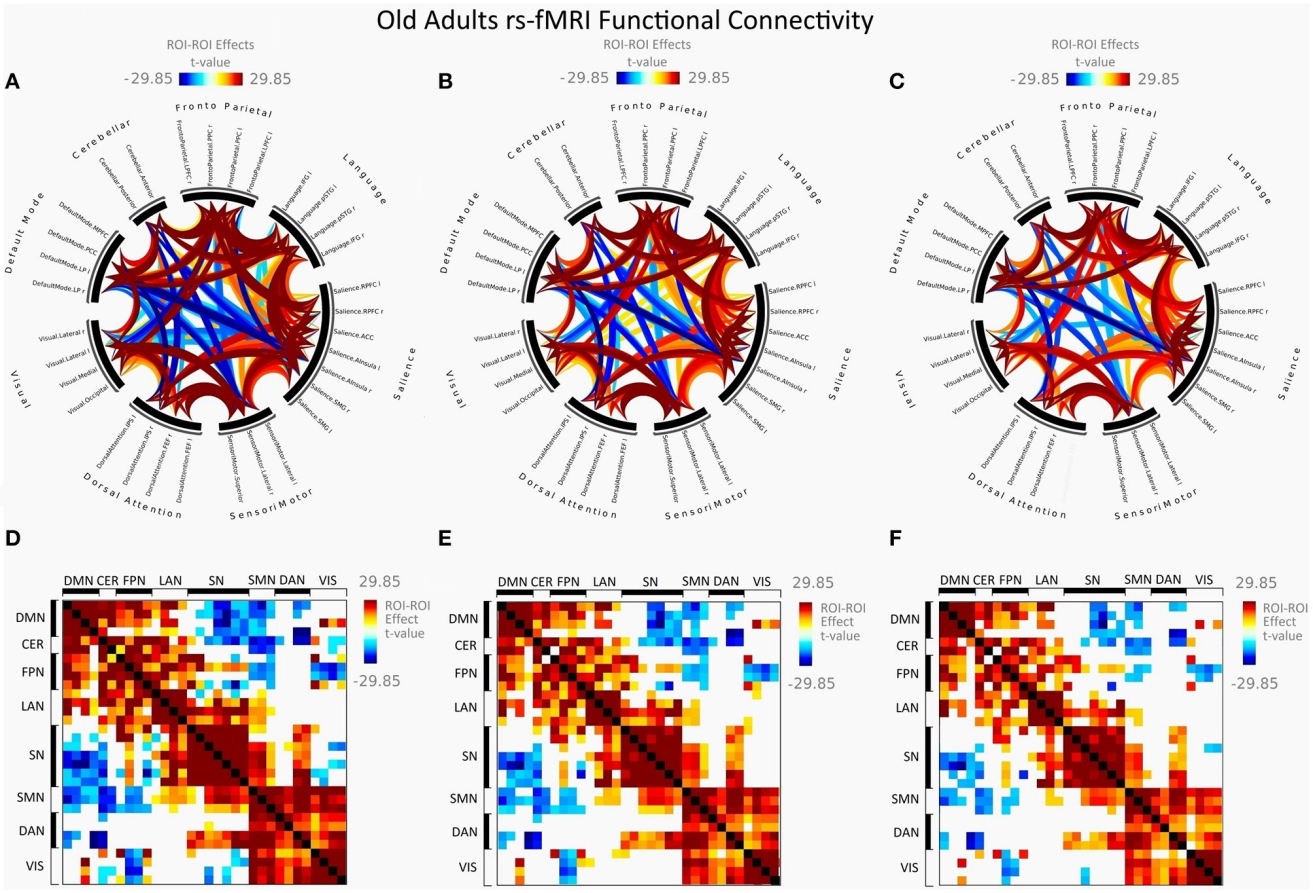
Mean FC in female elderly adults presented as a connectome ring and a connectivity matrix showing nodes with increased FC (red) and decreased FC (blue). Connectome ring network correlations computed using: **(A)** Functional network connectivity (FNC). **(B)** Spatial pairwise clustering (SPC). **(C)** Network-based statistics (NBS). Connectome matrix network correlations computed using: **(D)** Functional network connectivity (FNC). **(E)** Spatial pairwise clustering (SPC). **(F)** Network-based statistics (NBS).

**Figure 4:**
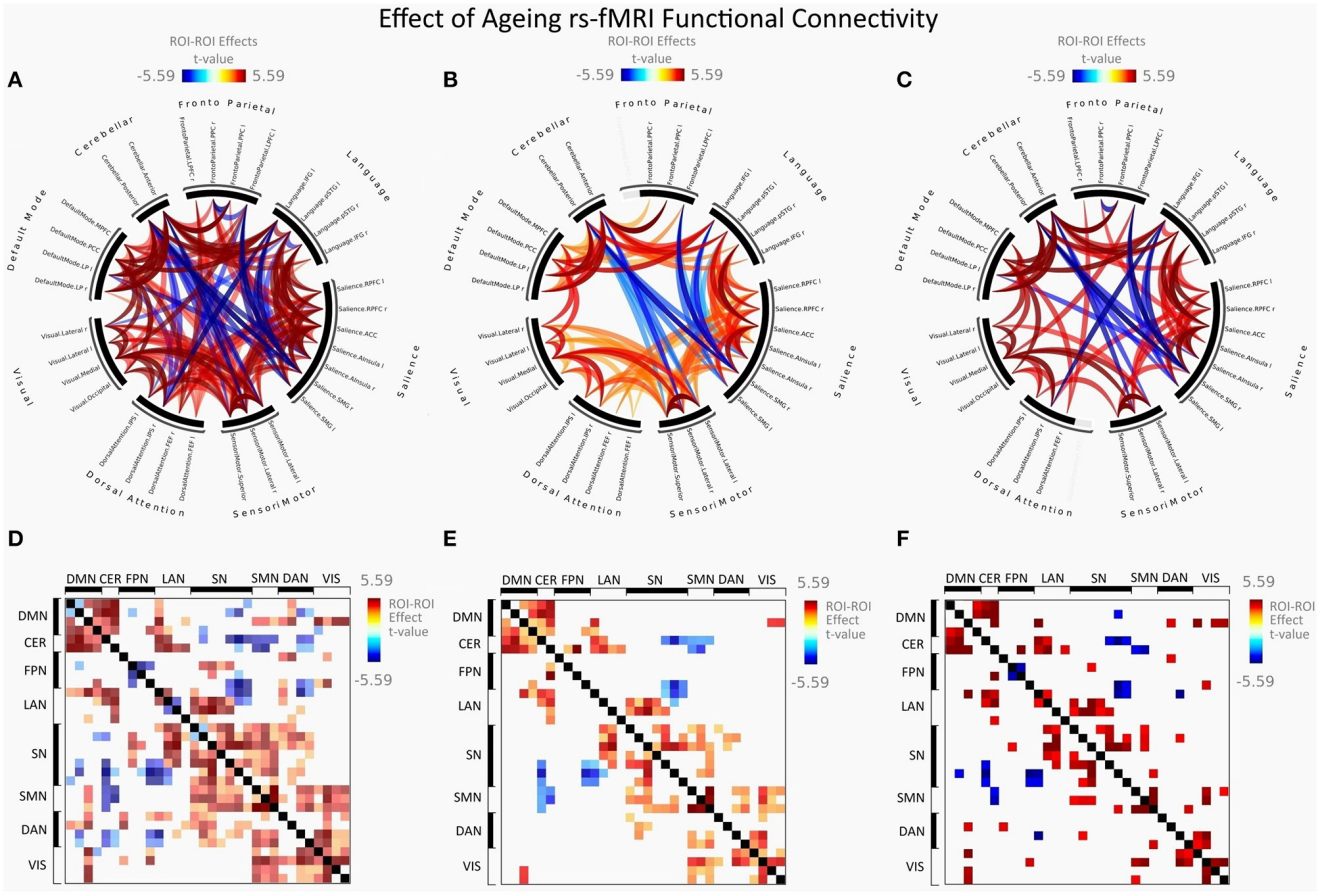
Group differences between young adults and older adults in FC presented as a connectome ring and connectivity a matrix showing nodes with increased FC (red) and decreased FC (blue). Connectome ring representation of: **(A)** Functional network connectivity (FNC). **(B)** Spatial pairwise clustering (SPC). **(C)** Network-based statistics (NBS). Connectome matrix representation of: **(D)** Functional network connectivity (FNC). **(E)** Spatial pairwise clustering (SPC). **(F)** Network-based statistics (NBS).

Figure 2 illustrates FC in younger females measured between rs-networks, while Figure 3 shows FNC in older women. The differences between groups in FC including eight main rs-networks are presented in Figure 4.

#### Increase in Intra-Network Brain Functional Connectivity Related to Older Age

There was a significant increase in the intra-network brain FC in older females (group 2) compared to young females (group 1) found in the following nodes of networks: the default mode (DMN), the salience (SN), the sensorimotor (SMN), and the visual (VIS) network.

**Figure 5:**
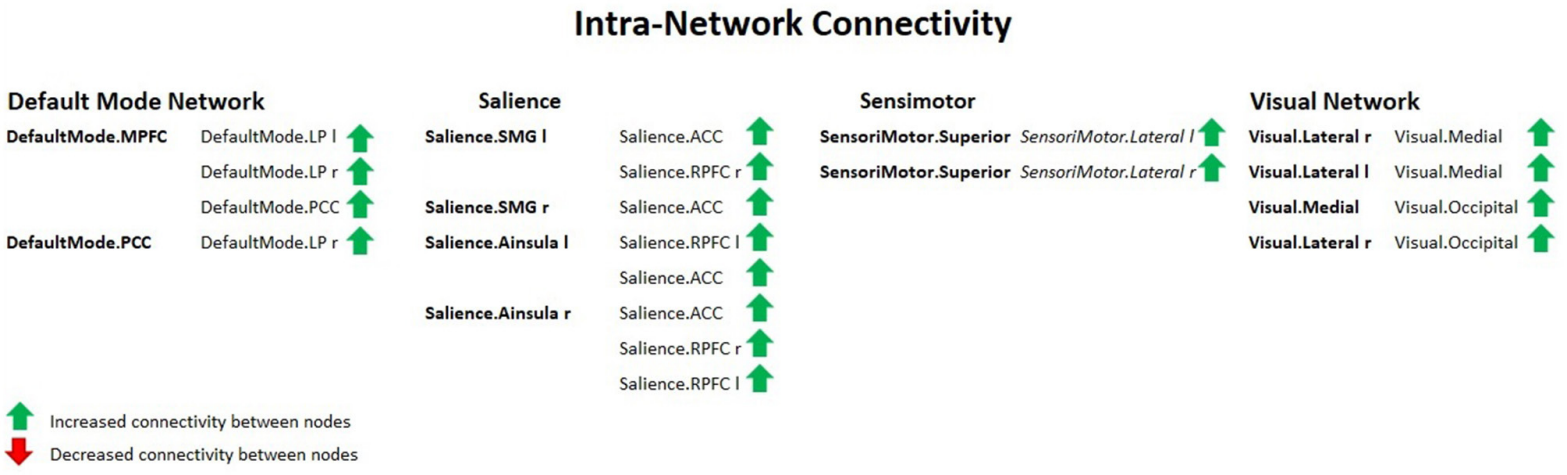
Intra-network connectivity table showing increased FC between nodes in females with ageing, using spatial pairwise clustering (SPC) method.

Figure 5 shows increased intra-network connectivity in females with ageing.

#### Increase in Inter-network Brain Functional Connectivity Related to Older Age

**Figure 6:**
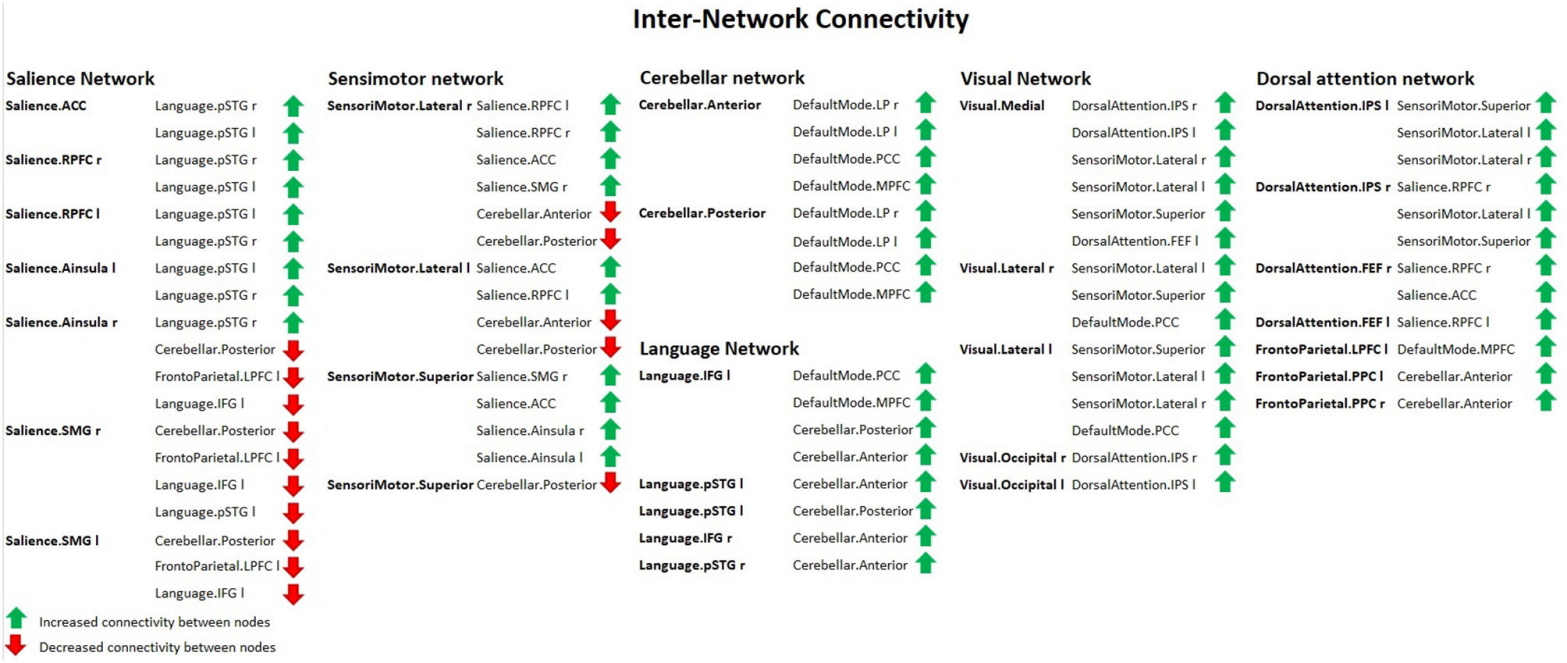
Inter-network connectivity table showing increased (green arrows) or decreased (red arrows) FC between nodes in females with ageing, using spatial pairwise clustering (SPC) method.

Group 2 (elderly females) presented a significantly increased **inter-network** brain FC bilaterally of sensorimotor lateral and salience rostral prefrontal cortex (RPFC), salience and language, salience and fronto-parietal, cerebellar anterior and default mode, cerebellar posterior and default mode, visual and sensorimotor lateral, visual lateral and default mode, language and cerebellar anterior, language and cerebellar posterior, fronto-parietal and cerebellar anterior, dorsal attention and sensorimotor, dorsal attention and default mode, and sensorimotor superior and salience networks (Figure 6).

Apart from that, the second group revealed a significant increase in the inter-network brain FC only on one side between: language inferior frontal gyrus (IFG) and right fronto-parietal posterior parietal cortex (PPC), left language IFG and default mode precuneus cortex (PCC), left language IFG and default mode medial prefrontal cortex (MPFC), right dorsal attention intraparietal sulcus (IPS), and right salience RPFC.

The results concerning significant increase in the intra- and inter-network brain FC are shown in Table 2.

#### Decrease in Inter-network Brain Functional Connectivity Related to Older Age

Compared to group 1 (female young adults), group 2 (female older adults) presented a significantly decreased inter-network brain FC bilaterally of the salience supramarginal gyrus (SMG) and the cerebellar posterior, the sensorimotor lateral and the cerebellar anterior networks, the sensorimotor lateral and the cerebellar posterior network, and the sensorimotor superior and cerebellar posterior network.

Additionally, group 2 showed a significant decrease in the inter-network brain FC only on one side between: dorsal attention IPS and cerebellar posterior, salience SMG–language IFG, right salience SMG and left fronto-parietal lateral prefrontal cortex (LPFC), and left salience SMG and left fronto-parietal LPFC.

The results concerning significant decrease in the inter-network brain FC are shown in Table 3 as well as in Figure 6.

#### Stabilisation in Inter-network Brain Functional Connectivity With Age

We found that some regions retained their FC characteristics with age. Several nodes were not significantly interconnected and remained the same as in the younger age. Those regions include such connexions as bilateral lateral parietal cortex–fronto-parietal posterior parietal cortex (PPC) bilateral as well as default mode bilateral lateral parietal cortex–salience rostral prefrontal cortex (RPFC) bilateral.

#### Comparison of the Results Obtained by Applying Various Approaches to Node Clustering and Thresholding: SPC vs. FNC, SPC vs. NBS, and NBS vs. SPC

Table 4 shows the significantly increased ROI–ROI FC in older women found in the SPC analysis in comparison to the FNC method in the networks: left fronto-parietal posterior parietal cortex (PPC) and cerebellar anterior as well as right fronto-parietal posterior parietal cortex (PPC) and cerebellar anterior.

**Table 4:** The increased functional connectivity in older females observed only in SPC analysis, not found in FNC method.

		SPC vs. FNC^*^		
		**T(58)**	***p*-FDR**	***p*-FWE**
FrontoParietal.PPC l	Cerebellar.Anterior	5.49	0.000001	0.000219
FrontoParietal.PPC r	Cerebellar.Anterior	3	0.004004	0.020124

* *Additional changes in connectivity observed in SPC and not presented as results in FNC analysis*.

Additionally, older women presented a significant increase in FC obtained with the SPC method and not observed in the NBS analysis between the following networks: cerebellar and default mode, sensorimotor and salience, visual and sensorimotor, dorsal attention and sensorimotor, dorsal attention, and salience as well as in the intra-network FC of the default mode, salience and VIS. A detailed comparison is shown in Table 5.

**Table 5:** The significant increase in functional connectivity in older females obtained only in SPC analysis, not found in NBS method.

		SPC vs. NBS^*^		
		**T(58)**	***p*-FDR**	***p*-FWE**
Cerebellar.Anterior	DefaultMode.MPFC	3.42	0.001157	0.003585
DefaultMode.PCC	DefaultMode.LP r	3.38	0.001319	0.005839
Cerebellar.Posterior	DefaultMode.PCC	3.15	0.002604	0.006727
Cerebellar.Posterior	DefaultMode.MPFC	2.83	0.006472	0.013375
DefaultMode.MPFC	DefaultMode.PCC	2.68	0.009627	0.042636
SensoriMotor.Superior	Salience.Ainsula r	3.46	0.001035	0.005826
SensoriMotor.Superior	Salience.Ainsula l	3.43	0.001128	0.005826
SensoriMotor.Lateral l	Salience.ACC	3.36	0.00139	0.006415
SensoriMotor.Lateral r	Salience.SMG r	3.3	0.001674	0.007412
SensoriMotor.Superior	Salience.ACC	3.16	0.002471	0.007661
Salience.Ainsula l	Salience.ACC	3.02	0.003759	0.023305
SensoriMotor.Lateral l	Salience.RPFC l	2.77	0.007616	0.021464
Salience.RPFC r	Language.pSTG l	2.76	0.0077	0.029838
FrontoParietal.LPFC l	DefaultMode.MPFC	3.41	0.001198	0.009284
Language.pSTG r	Cerebellar.Anterior	3.37	0.001355	0.007001
Language.IFG l	DefaultMode.MPFC	2.86	0.005848	0.022661
Visual.Medial	Visual.Occipital	3	0.004017	0.013006
Visual.Lateral r	Visual.Occipital	2.92	0.004989	0.030932
Visual.Medial	SensoriMotor.Superior	3.37	0.00134	0.005193
Visual.Lateral l	SensoriMotor.Lateral l	3.23	0.002058	0.021263
Visual.Lateral r	SensoriMotor.Superior	3.17	0.002415	0.018718
Visual.Lateral l	SensoriMotor.Superior	3.1	0.002995	0.023211
Visual.Medial	DorsalAttention.FEF l	2.97	0.004273	0.013006
Visual.Lateral l	SensoriMotor.Lateral r	2.73	0.008275	0.051306
DorsalAttention.IPS l	SensoriMotor.Lateral l	3.42	0.001153	0.008939
DorsalAttention.IPS r	SensoriMotor.Lateral l	3.3	0.001655	0.010264
DorsalAttention.IPS l	SensoriMotor.Lateral r	2.93	0.004861	0.025115
FrontoParietal.PPC r	Cerebellar.Anterior	3	0.004004	0.020124
DorsalAttention.FEF r	Salience.RPFC r	3.35	0.001412	0.014595
DorsalAttention.FEF r	Salience.ACC	2.88	0.005612	0.043496
DorsalAttention.FEF l	Salience.RPFC l	2.69	0.00941	0.05834

* *Additional changes in FC observed in SPC and not presented as results in NBS analysis*.

The significantly decreased FC between networks in group 2 observed in the SPC analysis but not in NBS are as follows: between salience and fronto-parietal as well as between salience and LAN (Table 6).

**Table 6:** The significant decrease in functional connectivity in older females observed only in SPC analysis, not revealed in NBS method.

		SPC vs. NBS^*^		
		**T(58)**	***p*-FDR**	***p*-FWE**
Salience.Ainsula r	FrontoParietal.LPFC l	−3.06	0.003366	0.011595
Salience.SMG r	Language.pSTG l	−3	0.004016	0.015564
Salience.Ainsula r	Language.IFG l	−2.73	0.008322	0.025797

* *Additional changes in FC observed in SPC and not present as results in NBS analysis*.

Older women showed a significantly increased inter-network FC with the NBS method, not found in the SPC analysis, between salience and fronto-parietal, dorsal attention and default mode, visual and fronto-parietal, and visual and default mode (Table 7).

**Table 7:** The increased functional connectivity in older women observed only in NBS analysis, not found in SPC method.

		NBS vs. SPC^*^		
		**T(58)**	***p*-FDR**	***p*-FWE**
Language.pSTG l	Language.IFG l	3.56	0.000746	0.00673
Salience.ACC	FrontoParietal.PPC r	3.49	0.000916	0.007294
DorsalAttention.FEF r	DefaultMode.PCC	3.8	0.000351	0.004045
Visual.Medial	FrontoParietal.LPFC l	3.88	0.000269	0.003295
Visual.Occipital	DefaultMode.PCC	4.53	0.00003	0.000735

* *Additional changes in FC observed in NBS and not presented as results in SPC analysis*.

Furthermore, the NBS analysis revealed a significantly decreased FC in older females between the dorsal attention and the LAN, the salience and the DMN as well as in the intra-network FC of the FPN, as shown in Table 8.

**Table 8:** The decreased functional connectivity in older women observed only in NBS analysis, not found in the SPC method.

		NBS vs. SPC^*^		
		**T(58)**	***p*-FDR**	***p*-FWE**
DorsalAttention.IPS r	Language.IFG l	−4.48	0.000035	0.000836
FrontoParietal.PPC l	FrontoParietal.PPC r	−4.19	0.000095	0.001478
Salience.SMG r	DefaultMode.LP l	−3.54	0.000785	0.006954

* *Additional changes in FC observed in NBS and not presented as results in SPC analysis*.

In summary, the FNC method presented the highest number of results, the NBS approach was the most restrictive one, while SPC analysis showed intermediate results with the exceptions presented in Tables 5–8, which may be explained by different statistical approaches to cluster definition and family-wise error control.

Detailed results obtained with the FNC (Supplementary Table 1), and NBS method (Supplementary Table 2) are available as Supplementary Materials.

## Discussion

Overall, most of the studies which assessed the ageing process with the use of rs-MRI reported age-related impairment in RSFC with a special focus on the DMN (10, 12–14, 31–38). The authors concluded that, in general, ageing is connected with decrease within the DMN. However, there have been articles showing both increase and decrement in RSFC related to older age (39). On the other hand, one study did not reveal any effects of ageing on RSFC in the DMN (40), but this research should not be taken into account as the study group comprised only elderly subjects without comparison to younger adults.

### Increase in Intra- and Inter-Network Brain FC With Ageing

In our study, we found a significant increase in the intra- and inter-network brain FC within the DMN in elderly females compared to the younger ones. We assume that the results could be associated with the method we used. Our analysis comprised networks of ROIs defined with the CONN network atlas. It should be stressed that the hippocampus region was not included within the detailed ROI assessment. Nevertheless, regarding the analysed networks, our results revealed that older women showed an increase compared with younger females in the intra- and inter-network brain FC within the DMN.

Apart from that, most of the reports published in the literature evaluated the age-related differences in RSFC without distinguishing between men and women. According to current science, little is known about sex differences in RSFC (41, 42). Zonneveld et al. (41) observed higher connectivity within the DMN as well as higher connectivity within the sensorimotor and VISs in females, which is in accordance with our findings.

Interestingly enough, ageing is accompanied by an increase in some of the processes that contribute to image processing in the brain. The integration of sensory information is optimised with ageing. Nerve networks in the brain restructure in response to the decrease of some sensorial functions (43, 44). Occipital activation by visual perception tends to decrease with age. In parallel to this, activation of the areas associated with control functions, particularly in the prefrontal cortex, increases (45). This reorganisation enables a compensatory recruitment of the structures that integrate the endogenous process of analysis (44).

We found increased intra-network as well as inter-network brain FC related to older age within the VIS in women. Our results are consistent with other authors' findings (41).

Increased activity within the VIS may indicate an increased functional involvement of cortical structures in this area. This may be indicative of a compensatory mechanism associated with the physiological impairment of light signal perception and a decrease in the density of neurons in the retina and lateral geniculate bodies during ageing.

### Age-Related Increase in FC in Short-Range Connexions

Moreover, we observed an age-related increase in RSFC in short-range connexions such as between the nodes of left FPN lateral prefrontal cortex–DMN medial prefrontal cortex as well as the right DAN frontal eye field–DMN precuneus cortex. Similar results have also been reported in only one study (41). These findings seem to suggest that in older subjects RSFC increases between networks that show close anatomical proximity in the brain, while it decreases with age between networks that are located far away from each other in the brain. To the best of our knowledge, this is the second study indicating such a mechanism associated with the ageing process of the brain.

### Increased Functional Activity of the Cerebellum in Older Women

Another interesting concept is related to cerebellar function which may lead to the hypothesis that the cerebellum could play a more important role in healthy ageing than previously supposed and can be assessed with the use of structural MRI.

The cerebellum, thanks to numerous centripetal and centrifugal connexion pathways through the nuclei of the brainstem and the reticular system, plays a key role in automated regulatory processes that ensure maintaining balance, counteracting gravity, and coordinating deliberate voluntary movements and muscle tension (46). The cerebellum is also most likely involved in regulating cognitive and behavioural functions through its interactions with different cortical regions in different connectivity networks (47, 48). Several studies support the thesis that the cerebellum acts in cognition in the same fashion as cerebro-cerebellar connexions organised into long-range loops (46, 49). Previous studies on the structural characteristics of the human cerebellum were conducted in the context of a specific pathology or concerned a limited age range. In fact, research into life-long maturation and ageing of the cerebellum is rare, and most have considered the cerebellum as a whole without examining each lobule.

It seems that the increased activity of the cerebellum in older women may be related to the mechanism of recruiting more brain volume for cognitive purposes with age. The cerebellum most likely activates its long-range connexions with the rest of the cortical regions, including the prefrontal cortex, but it does not increase the speed of information transmission (46). Therefore, our data suggest that the cerebellum may be resistant to certain neurodegenerative mechanisms. To the best of our knowledge, our study is the first to present the importance of cerebellar network increase during healthy brain ageing in women.

### The Possible Mechanisms Associated With the Increased FC With Ageing

We assume that the increased FC within some of the networks may reflect a compensatory mechanism; however, the increase could also be explained by neuronal excitotoxicity.

Neuronal excitotoxicity usually refers to the injury and death of neurons arising from prolonged exposure to glutamate which is the major excitatory neurotransmitter in human central nervous system. Excitotoxicity could be regarded also as a consequence of other cellular phenomena, such as mitochondrial dysfunction, physical neuronal damage, and oxidative stress (50). However, extracellular concentrations of glutamate in the human brain may also increase progressively with normal ageing (51).

Age-related changes may be caused by disruption of myelinated fibres that connect neurons in different cortical regions (52). In normal ageing, brain changes in the synaptic physiology of ageing neurons may contribute to altered connectivity (53). This process may contribute to compensatory increased neural activity in the older individuals, which could predispose the individuals to excitotoxicity and neurodegenerative pathology (54).

### Limitations

There are some limitations to our study. First of all, we included a relatively small sample size, besides we evaluated RSFC changes only in women. Obviously, further studies are needed in order to compare age-related alterations in RSFC in men and women as well as to assess the ageing process involving the RSNs only in men. Nevertheless, the fact that our analysis was restricted only to women can be considered as a strength of this study. It has been suggested that the male and female brains age in a different way. According to this thesis, men and women should be evaluated separately in order to avoid any impact of sex on age-related RSFC changes. Although we started our research with female subjects, obviously we are going to study the intra- and inter-network RSFC differences between young and older male brains in the future as well.

Furthermore, our analysis was limited to eight main RSNs and main network nodes within each analysed network. However, we evaluated the cerebellar network that has often been omitted in other papers.

### Strengths of the Study

On the other hand, it should be stressed that our study was performed using a modern 3-Tesla MR scanner equipped with gradients (45 mT and 200 T/m/s slew rate) with parallel and multislice acceleration and a 32-channel head coil resulting in a high signal-to-noise ratio achieved together with a relatively high spatial and temporal resolution of the acquired data.

In our study, time of scanning was optimised in order to achieve high intersession and intrasession reliability (55). Furthermore, the spatial resolution was a trade-off between SNR and temporal resolution (56).

Unfortunately, we cannot exactly quantify how short TR acquisition improved our results because that was not our null hypothesis. However, the effects of spatial resolution, increased acquisition time, and short TR on BOLD data have been widely discussed in the literature. Multislice rs-fMRI scan has improved the signal-to-noise ratio, while preserving the same level of the test–retest reliability compared to conventional EPI (57).

Most widely reported in the literature benefits of short TRs on stationary rs-fMRI metrics are minimal when compared with dynamic connectivity metrics. The analysis of dynamic connectivity should benefit more from shorter TRs than stationary metrics (58, 59).

We could only hypothesise that acquiring 800 timepoints and using multislice short TR acquisition improved our sensitivity and specificity similarly to the event-related fMRI studies available in the literature (60).

Moreover, we conducted the study using three different approaches to connectivity analysis. In our work, the main method of analysis was the ROI-ROI SPC (30). We also presented additional results obtained from different procedures such as FNC (27) and NBS (29). This approach allowed not only to reveal differences between the methods that we used but also to confirm our results within three different approaches used in the literature.

In summary, the results of our study seem to suggest that with older age, the brain appears to undergo a complicated reorganisation process associated with the integration and segregation of large-scale rs-networks. This assumption has also been proposed in other reports available in the literature (41, 61, 62).

Overall, our findings suggest that RSFC changes in brain networks can serve as sensitive biomarkers for the age-related changes which are invisible in the structural MRI. Our results could support the idea that during ageing some inter-network connexions may function as a compensating mechanism to age-related FC changes.

## Conclusions

Our study seems to indicate a reorganisation mechanism of large-scale functional brain networks during healthy female ageing, and it supports the evidence that apart from an overall reduction in RSFC between main rs-networks, ageing in women can lead to substantially increased FC between nodes of the default mode, the salience, the sensorimotor, the language, the fronto-parietal, the dorsal attention, and the VIS as well as the cerebellar anterior and cerebellar posterior networks.

It should be stressed that most of the reports on age-related changes in the brain observed with the rs-fMRI studies concentrated on RSFC alterations within large-scale RSNs, except for the cerebellar network (CER). We assessed eight large-scale RSNs including the CER network. To the best of our knowledge, this study is the first to report the importance of cerebellar network increase during healthy ageing in women.

Moreover, little is known about age-related increase in RSFC within the VIS. We observed increased RSFC related to older age within the VIS in women and we also attempted to discuss the possible mechanisms responsible for these findings.

We believe that this project will contribute new knowledge to the comprehension of brain ageing while in the future our results could influence and improve the diagnosis and treatment of cognitive disorders in elderly people.

## Data Availability Statement

The original contributions presented in the study are included in the article/Supplementary Material, further inquiries can be directed to the corresponding author.

## Ethics Statement

The studies involving human participants were reviewed and approved by the Wroclaw Medical University Ethics Committee for conducting research involving humans no. of permission KB-57/2021. The patients/participants provided their written informed consent to participate in this study.

## Author Contributions

PP analyzed and interpreted rs-fMRI data, performed all the figures and tables, conducted literature search, and wrote the manuscript. MW-P contributed to the study design, wrote some parts of the manuscript, conducted literature search, and critically reviewed the paper. AZ contributed to the study design and critically reviewed the manuscript. MS contributed to the study design and critically reviewed the paper. JB, contributed to the study design, wrote some parts of the manuscript, supervised writing of the manuscript, critically reviewed the paper and supervised the research project. All authors contributed to the article and approved the submitted version.

## Conflict of Interest

The authors declare that the research was conducted in the absence of any commercial or financial relationships that could be construed as a potential conflict of interest.
