# Supplementary material for: Resting-State Functional Connectivity of the Ageing Female Brain—Differences Between Young and Elderly Female Adults on Multislice Short TR rs-fMRI
Source: Front Neurol. 2021 Jul 12;12:645974. doi: 10.3389/fneur.2021.645974 (PMC8311596; doi:10.3389/fneur.2021.645974)
Supplement: Supplementary file 1 [file Table_1.docx]

**Supplementary Table 1.** Difference between Young Adults vs Old Adults calculated using Functional Network Connectivity (FNC) method.

| **Functional Network Connectivity** | | | | | | | |
| --- | --- | --- | --- | --- | --- | --- | --- |
|  | | | | | | | |
|  |  |  |  |  |  |  |  |
| **Analysis** |  |  | **Statistic** |  | **p-unc** | **p-FDR** | **p-FWE** |
|  |  |  |  |  |  |  |  |
| **Cluster 1** |  |  | **F(2,57) =** | **25.07** | **0** | **0.000001** |  |
| Connection | Salience.ACC | Language.pSTG r | T(58) = | 5.32 |  | 0.000002 | 0.000055 |
| Connection | Salience.ACC | Language.pSTG l | T(58) = | 4.75 |  | 0.000014 | 0.000108 |
| Connection | Salience.SMG r | Language.IFG l | T(58) = | -4.33 |  | 0.00006 | 0.000465 |
| Connection | Salience.RPFC r | Language.pSTG r | T(58) = | 4.7 |  | 0.000016 | 0.000507 |
| Connection | Salience.Ainsula r | Language.pSTG r | T(58) = | 3.88 |  | 0.000272 | 0.002111 |
| Connection | Salience.RPFC l | Language.pSTG l | T(58) = | 4 |  | 0.00018 | 0.002798 |
| Connection | Salience.SMG l | Language.IFG l | T(58) = | -3.68 |  | 0.000518 | 0.005727 |
| Connection | Salience.RPFC l | Language.pSTG r | T(58) = | 3.52 |  | 0.00086 | 0.005817 |
| Connection | Salience.Ainsula l | Language.pSTG l | T(58) = | 3.92 |  | 0.000236 | 0.007326 |
| Connection | Salience.Ainsula l | Language.pSTG r | T(58) = | 3.47 |  | 0.000998 | 0.008739 |
| Connection | Salience.SMG r | Language.pSTG l | T(58) = | -3 |  | 0.004016 | 0.015564 |
| Connection | Salience.Ainsula r | Language.IFG l | T(58) = | -2.73 |  | 0.008322 | 0.025797 |
| Connection | Salience.RPFC r | Language.pSTG l | T(58) = | 2.76 |  | 0.0077 | 0.029838 |
| Connection | Salience.Ainsula r | Language.pSTG l | T(58) = | 2.43 |  | 0.018374 | 0.04342 |
| Connection | Salience.RPFC l | Language.IFG l | T(58) = | 2.43 |  | 0.018005 | 0.050742 |
| Connection | Salience.SMG l | Language.pSTG l | T(58) = | -2.47 |  | 0.016404 | 0.065873 |
| Connection | Salience.RPFC r | Language.IFG r | T(58) = | 2.16 |  | 0.03517 | 0.070496 |
|  |  |  |  |  |  |  |  |
| **Cluster 2** |  |  | **F(2,57) =** | **21.09** | **0** | **0.000003** |  |
| Connection | Salience.SMG r | Salience.ACC | T(58) = | 5.07 |  | 0.000004 | 0.000083 |
| Connection | Salience.Ainsula r | Salience.ACC | T(58) = | 4.93 |  | 0.000007 | 0.000224 |
| Connection | Salience.Ainsula r | Salience.RPFC r | T(58) = | 4.2 |  | 0.000094 | 0.001455 |
| Connection | Salience.Ainsula r | Salience.RPFC l | T(58) = | 4.07 |  | 0.000144 | 0.001489 |
| Connection | Salience.SMG l | Salience.ACC | T(58) = | 4.36 |  | 0.000054 | 0.001684 |
| Connection | Salience.SMG l | Salience.RPFC r | T(58) = | 3.48 |  | 0.000965 | 0.005984 |
| Connection | Salience.SMG l | Salience.Ainsula r | T(58) = | 3.29 |  | 0.001688 | 0.008723 |
| Connection | Salience.Ainsula l | Salience.RPFC l | T(58) = | 3.49 |  | 0.000938 | 0.008739 |
| Connection | Salience.Ainsula l | Salience.ACC | T(58) = | 3.02 |  | 0.003759 | 0.023305 |
| Connection | Salience.ACC | Salience.RPFC l | T(58) = | 2.49 |  | 0.015683 | 0.034727 |
| Connection | Salience.SMG r | Salience.Ainsula r | T(58) = | 2.55 |  | 0.013477 | 0.046422 |
| Connection | Salience.SMG l | Salience.RPFC l | T(58) = | 2.46 |  | 0.017 | 0.065873 |
| Connection | Salience.Ainsula l | Salience.RPFC r | T(58) = | 2.48 |  | 0.016108 | 0.071334 |
| Connection | Salience.RPFC r | Salience.RPFC l | T(58) = | -2.07 |  | 0.043246 | 0.077461 |
| Connection | Salience.SMG r | Salience.RPFC r | T(58) = | 2.14 |  | 0.036385 | 0.086765 |
| Connection | Salience.SMG l | Salience.Ainsula l | T(58) = | 2.08 |  | 0.041629 | 0.107541 |
|  |  |  |  |  |  |  |  |
| **Cluster 3** |  |  | **F(2,57) =** | **19.45** | **0** | **0.000004** |  |
| Connection | Cerebellar.Anterior | DefaultMode.LP r | T(58) = | 5.13 |  | 0.000004 | 0.000045 |
| Connection | Cerebellar.Anterior | DefaultMode.LP l | T(58) = | 5.07 |  | 0.000004 | 0.000045 |
| Connection | Cerebellar.Posterior | DefaultMode.LP l | T(58) = | 5.23 |  | 0.000002 | 0.000075 |
| Connection | Cerebellar.Anterior | DefaultMode.PCC | T(58) = | 4.61 |  | 0.000023 | 0.000141 |
| Connection | Cerebellar.Posterior | DefaultMode.LP r | T(58) = | 4.34 |  | 0.000058 | 0.000451 |
| Connection | Cerebellar.Anterior | DefaultMode.MPFC | T(58) = | 3.42 |  | 0.001157 | 0.003585 |
| Connection | Cerebellar.Posterior | DefaultMode.PCC | T(58) = | 3.15 |  | 0.002604 | 0.006727 |
| Connection | Cerebellar.Posterior | DefaultMode.MPFC | T(58) = | 2.83 |  | 0.006472 | 0.013375 |
|  |  |  |  |  |  |  |  |
| **Cluster 4** |  |  | **F(2,57) =** | **16.12** | **0** | **0.000026** |  |
| Connection | Language.pSTG l | Cerebellar.Anterior | T(58) = | 4.62 |  | 0.000022 | 0.000334 |
| Connection | Language.IFG l | Cerebellar.Posterior | T(58) = | 4.41 |  | 0.000045 | 0.000464 |
| Connection | Language.IFG l | Cerebellar.Anterior | T(58) = | 4.03 |  | 0.000164 | 0.001016 |
| Connection | Language.pSTG l | Cerebellar.Posterior | T(58) = | 3.73 |  | 0.000441 | 0.002735 |
| Connection | Language.pSTG r | Cerebellar.Anterior | T(58) = | 3.37 |  | 0.001355 | 0.007001 |
| Connection | Language.IFG r | Cerebellar.Anterior | T(58) = | 3.67 |  | 0.000526 | 0.016313 |
|  |  |  |  |  |  |  |  |
| **Cluster 5** |  |  | **F(2,57) =** | **13.34** | **0** | **0.000128** |  |
| Connection | DefaultMode.MPFC | DefaultMode.LP l | T(58) = | 4.2 |  | 0.000095 | 0.002932 |
| Connection | DefaultMode.PCC | DefaultMode.LP r | T(58) = | 3.38 |  | 0.001319 | 0.005839 |
| Connection | DefaultMode.MPFC | DefaultMode.LP r | T(58) = | 3.72 |  | 0.000451 | 0.00699 |
| Connection | DefaultMode.MPFC | DefaultMode.PCC | T(58) = | 2.68 |  | 0.009627 | 0.042636 |
| Connection | DefaultMode.LP l | DefaultMode.LP r | T(58) = | -2.08 |  | 0.042064 | 0.121206 |
|  |  |  |  |  |  |  |  |
| **Cluster 6** |  |  | **F(2,57) =** | **12.09** | **0** | **0.000251** |  |
| Connection | Visual.Occipital | DefaultMode.PCC | T(58) = | 4.53 |  | 0.00003 | 0.000919 |
| Connection | Visual.Lateral r | DefaultMode.PCC | T(58) = | 4.28 |  | 0.000071 | 0.001094 |
| Connection | Visual.Lateral l | DefaultMode.PCC | T(58) = | 4.2 |  | 0.000093 | 0.001436 |
| Connection | Visual.Medial | DefaultMode.PCC | T(58) = | 2.54 |  | 0.013714 | 0.032702 |
| Connection | Visual.Occipital | DefaultMode.MPFC | T(58) = | -2.34 |  | 0.02262 | 0.100176 |
|  |  |  |  |  |  |  |  |
| **Cluster 7** |  |  | **F(2,58) =** | **16.79** | **0** | **0.000585** |  |
| Connection | SensoriMotor.Superior | SensoriMotor.Lateral l | T(58) = | 5.59 |  | 0.000001 | 0.00002 |
| Connection | SensoriMotor.Superior | SensoriMotor.Lateral r | T(58) = | 5.39 |  | 0.000001 | 0.000021 |
| Connection | SensoriMotor.Lateral r | SensoriMotor.Lateral l | T(58) = | 2.06 |  | 0.043785 | 0.067866 |
|  |  |  |  |  |  |  |  |
| **Cluster 8** |  |  | **F(2,57) =** | **10.36** | **0** | **0.000585** |  |
| Connection | Language.pSTG l | Language.IFG l | T(58) = | 3.56 |  | 0.000746 | 0.003856 |
| Connection | Language.pSTG r | Language.pSTG l | T(58) = | -3.15 |  | 0.002615 | 0.011581 |
|  |  |  |  |  |  |  |  |
| **Cluster 9** |  |  | **F(2,57) =** | **10.35** | **0** | **0.000585** |  |
| Connection | Visual.Lateral r | Visual.Medial | T(58) = | 4.64 |  | 0.00002 | 0.000624 |
| Connection | Visual.Lateral l | Visual.Medial | T(58) = | 4.41 |  | 0.000045 | 0.001399 |
| Connection | Visual.Medial | Visual.Occipital | T(58) = | 3 |  | 0.004017 | 0.013006 |
| Connection | Visual.Lateral r | Visual.Occipital | T(58) = | 2.92 |  | 0.004989 | 0.030932 |
| Connection | Visual.Lateral l | Visual.Occipital | T(58) = | 2.06 |  | 0.043889 | 0.165215 |
|  |  |  |  |  |  |  |  |
| **Cluster 10** |  |  | **F(2,57) =** | **9.45** | **0** | **0.001027** |  |
| Connection | Salience.SMG r | FrontoParietal.LPFC l | T(58) = | -4.9 |  | 0.000008 | 0.000083 |
| Connection | Salience.ACC | FrontoParietal.PPC r | T(58) = | 3.49 |  | 0.000916 | 0.004056 |
| Connection | Salience.SMG l | FrontoParietal.LPFC l | T(58) = | -3.66 |  | 0.000554 | 0.005727 |
| Connection | Salience.RPFC r | FrontoParietal.PPC r | T(58) = | 3.17 |  | 0.002445 | 0.010827 |
| Connection | Salience.Ainsula r | FrontoParietal.PPC r | T(58) = | 3.11 |  | 0.002898 | 0.011231 |
| Connection | Salience.Ainsula r | FrontoParietal.LPFC l | T(58) = | -3.06 |  | 0.003366 | 0.011595 |
| Connection | Salience.ACC | FrontoParietal.PPC l | T(58) = | 2.81 |  | 0.006746 | 0.017428 |
| Connection | Salience.Ainsula l | FrontoParietal.PPC r | T(58) = | 2.34 |  | 0.022749 | 0.088154 |
| Connection | Salience.SMG r | FrontoParietal.PPC l | T(58) = | -2.05 |  | 0.045313 | 0.094555 |
|  |  |  |  |  |  |  |  |
| **Cluster 11** |  |  | **F(2,57) =** | **8.81** | **0** | **0.001516** |  |
| Connection | SensoriMotor.Lateral r | Cerebellar.Anterior | T(58) = | -4.14 |  | 0.000113 | 0.001165 |
| Connection | SensoriMotor.Lateral l | Cerebellar.Anterior | T(58) = | -3.58 |  | 0.000704 | 0.006415 |
| Connection | SensoriMotor.Lateral l | Cerebellar.Posterior | T(58) = | -3.31 |  | 0.001629 | 0.006415 |
| Connection | SensoriMotor.Superior | Cerebellar.Posterior | T(58) = | -3.26 |  | 0.001873 | 0.007258 |
| Connection | SensoriMotor.Lateral r | Cerebellar.Posterior | T(58) = | -3.23 |  | 0.00205 | 0.007943 |
| **Cluster 12** |  |  | **F(2,57) =** | **8.65** | **0** | **0.001569** |  |
| Connection | DorsalAttention.FEF r | DefaultMode.PCC | T(58) = | 3.8 |  | 0.000351 | 0.007961 |
| Connection | DorsalAttention.IPS r | DefaultMode.LP l | T(58) = | -3.06 |  | 0.003393 | 0.013148 |
| Connection | DorsalAttention.FEF l | DefaultMode.PCC | T(58) = | 2.73 |  | 0.008335 | 0.05834 |
| Connection | DorsalAttention.FEF r | DefaultMode.LP r | T(58) = | 2.6 |  | 0.011889 | 0.061159 |
| Connection | DorsalAttention.FEF r | DefaultMode.LP l | T(58) = | 2.16 |  | 0.034753 | 0.097118 |
| Connection | DorsalAttention.IPS l | DefaultMode.LP l | T(58) = | -2.07 |  | 0.043008 | 0.099638 |
|  |  |  |  |  |  |  |  |
| **Cluster 13** |  |  | **F(2,57) =** | **8.43** | **0** | **0.00172** |  |
| Connection | Salience.SMG r | Cerebellar.Posterior | T(58) = | -4.95 |  | 0.000007 | 0.000083 |
| Connection | Salience.Ainsula r | Cerebellar.Posterior | T(58) = | -3.69 |  | 0.000496 | 0.003073 |
| Connection | Salience.SMG l | Cerebellar.Posterior | T(58) = | -3.54 |  | 0.000803 | 0.005984 |
| Connection | Salience.RPFC r | Cerebellar.Posterior | T(58) = | -2.65 |  | 0.010342 | 0.035621 |
| Connection | Salience.SMG r | Cerebellar.Anterior | T(58) = | -2.04 |  | 0.045753 | 0.094555 |
|  |  |  |  |  |  |  |  |
| **Cluster 14** |  |  | **F(2,57) =** | **8.31** | **0** | **0.001749** |  |
| Connection | SensoriMotor.Lateral r | Salience.RPFC | T(58) = | 3.75 |  | 0.000413 | 0.002669 |
| Connection | SensoriMotor.Lateral r | Salience.ACC | T(58) = | 3.73 |  | 0.000431 | 0.002669 |
| Connection | SensoriMotor.Superior | Salience.SMG r | T(58) = | 3.68 |  | 0.000507 | 0.003929 |
| Connection | SensoriMotor.Lateral r | Salience.RPFC l | T(58) = | 3.51 |  | 0.000876 | 0.004524 |
| Connection | SensoriMotor.Superior | Salience.Ainsula r | T(58) = | 3.46 |  | 0.001035 | 0.005826 |
| Connection | SensoriMotor.Superior | Salience.Ainsula l | T(58) = | 3.43 |  | 0.001128 | 0.005826 |
| Connection | SensoriMotor.Lateral l | Salience.ACC | T(58) = | 3.36 |  | 0.00139 | 0.006415 |
| Connection | SensoriMotor.Lateral r | Salience.SMG r | T(58) = | 3.3 |  | 0.001674 | 0.007412 |
| Connection | SensoriMotor.Superior | Salience.ACC | T(58) = | 3.16 |  | 0.002471 | 0.007661 |
| Connection | SensoriMotor.Lateral l | Salience.RPFC l | T(58) = | 2.77 |  | 0.007616 | 0.021464 |
| Connection | SensoriMotor.Lateral r | Salience.Ainsula r | T(58) = | 2.54 |  | 0.013628 | 0.028165 |
| Connection | SensoriMotor.Lateral r | Salience.Ainsula l | T(58) = | 2.5 |  | 0.015259 | 0.029563 |
| Connection | SensoriMotor.Lateral l | Salience.RPFC r | T(58) = | 2.61 |  | 0.01153 | 0.029786 |
| Connection | SensoriMotor.Lateral l | Salience.Ainsula r | T(58) = | 2.3 |  | 0.024838 | 0.054999 |
| Connection | SensoriMotor.Lateral l | Salience.SMG r | T(58) = | 2.24 |  | 0.028836 | 0.059594 |
| Connection | SensoriMotor.Superior | Salience.SMG l | T(58) = | 2.18 |  | 0.033601 | 0.063106 |
| Connection | SensoriMotor.Superior | Salience.RPFC l | T(58) = | 2.12 |  | 0.038678 | 0.063106 |
| Connection | SensoriMotor.Lateral l | Salience.Ainsula l | T(58) = | 2.09 |  | 0.040565 | 0.073972 |
| Connection | SensoriMotor.Superior | Salience.RPFC r | T(58) = | 2.01 |  | 0.048736 | 0.075541 |
|  |  |  |  |  |  |  |  |
| **Cluster 15** |  |  | **F(2,57) =** | **7.28** | **0** | **0.003669** |  |
| Connection | DorsalAttention.IPS r | Language.IFG l | T(58) = | -4.48 |  | 0.000035 | 0.000549 |
| Connection | DorsalAttention.FEF l | Language.IFG l | T(58) = | 2.82 |  | 0.006625 | 0.05834 |
| Connection | DorsalAttention.IPS l | Language.IFG l | T(58) = | -2.49 |  | 0.015807 | 0.061252 |
| Connection | DorsalAttention.IPS r | Language.pSTG r | T(58) = | 2.11 |  | 0.039048 | 0.075655 |
| Connection | DorsalAttention.FEF l | Language.pSTG l | T(58) = | 2.43 |  | 0.018191 | 0.08056 |
| Connection | DorsalAttention.FEF r | Language.IFG r | T(58) = | 2.18 |  | 0.03308 | 0.097118 |
|  |  |  |  |  |  |  |  |
| **Cluster 16** |  |  | **F(2,57) =** | **7.09** | **0** | **0.003998** |  |
| Connection | DorsalAttention.IPS l | SensoriMotor.Superior | T(58) = | 3.85 |  | 0.000293 | 0.004541 |
| Connection | DorsalAttention.IPS l | SensoriMotor.Lateral l | T(58) = | 3.42 |  | 0.001153 | 0.008939 |
| Connection | DorsalAttention.IPS r | SensoriMotor.Lateral l | T(58) = | 3.3 |  | 0.001655 | 0.010264 |
| Connection | DorsalAttention.IPS r | SensoriMotor.Superior | T(58) = | 2.98 |  | 0.004248 | 0.014632 |
| Connection | DorsalAttention.IPS l | SensoriMotor.Lateral r | T(58) = | 2.93 |  | 0.004861 | 0.025115 |
| Connection | DorsalAttention.IPS r | SensoriMotor.Lateral r | T(58) = | 2.56 |  | 0.012996 | 0.040288 |
| Connection | DorsalAttention.FEF l | SensoriMotor.Lateral r | T(58) = | 2.29 |  | 0.025978 | 0.08948 |
|  |  |  |  |  |  |  |  |
| **Cluster 17** |  |  | **F(2,57) =** | **6.81** | **0** | **0.004581** |  |
| Connection | DorsalAttention.IPS r | Salience.RPFC r | T(58) = | 3.5 |  | 0.000891 | 0.006907 |
| Connection | DorsalAttention.FEF r | Salience.RPFC r | T(58) = | 3.35 |  | 0.001412 | 0.014595 |
| Connection | DorsalAttention.FEF r | Salience.ACC | T(58) = | 2.88 |  | 0.005612 | 0.043496 |
| Connection | DorsalAttention.IPS r | Salience.SMG r | T(58) = | 2.45 |  | 0.017329 | 0.048835 |
| Connection | DorsalAttention.IPS l | Salience.ACC | T(58) = | 2.57 |  | 0.012742 | 0.056431 |
| Connection | DorsalAttention.FEF l | Salience.RPFC l | T(58) = | 2.69 |  | 0.00941 | 0.05834 |
| Connection | DorsalAttention.FEF r | Salience.RPFC l | T(58) = | 2.54 |  | 0.01381 | 0.061159 |
| Connection | DorsalAttention.IPS r | Salience.ACC | T(58) = | 2.27 |  | 0.026944 | 0.068782 |
| Connection | DorsalAttention.IPS r | Salience.Ainsula r | T(58) = | 2.04 |  | 0.045479 | 0.082933 |
| Connection | DorsalAttention.FEF l | Salience.RPFC r | T(58) = | 2.36 |  | 0.021903 | 0.084874 |
| Connection | DorsalAttention.FEF r | Salience.SMG r | T(58) = | 2.29 |  | 0.025693 | 0.095684 |
| Connection | DorsalAttention.FEF r | Salience.SMG l | T(58) = | 2.26 |  | 0.027779 | 0.095684 |
| Connection | DorsalAttention.IPS l | Salience.RPFC r | T(58) = | 2.19 |  | 0.032854 | 0.09832 |
| Connection | DorsalAttention.IPS l | Salience.Ainsula l | T(58) = | 2.01 |  | 0.049543 | 0.099638 |
| Connection | DorsalAttention.FEF l | Salience.ACC | T(58) = | 2.01 |  | 0.049487 | 0.120269 |
|  |  |  |  |  |  |  |  |
| **Cluster 18** |  |  | **F(2,57) =** | **6.78** | **0** | **0.004581** |  |
| Connection | Visual.Medial | SensoriMotor.Lateral r | T(58) = | 4.57 |  | 0.000026 | 0.000271 |
| Connection | Visual.Medial | SensoriMotor.Lateral l | T(58) = | 4.22 |  | 0.000086 | 0.000535 |
| Connection | Visual.Medial | SensoriMotor.Superior | T(58) = | 3.37 |  | 0.00134 | 0.005193 |
| Connection | Visual.Lateral r | SensoriMotor.Lateral l | T(58) = | 3.49 |  | 0.000941 | 0.009725 |
| Connection | Visual.Lateral r | SensoriMotor.Superior | T(58) = | 3.17 |  | 0.002415 | 0.018718 |
| Connection | Visual.Lateral l | SensoriMotor.Lateral l | T(58) = | 3.23 |  | 0.002058 | 0.021263 |
| Connection | Visual.Lateral l | SensoriMotor.Superior | T(58) = | 3.1 |  | 0.002995 | 0.023211 |
| Connection | Visual.Lateral r | SensoriMotor.Lateral r | T(58) = | 2.62 |  | 0.011236 | 0.049759 |
| Connection | Visual.Lateral l | SensoriMotor.Lateral r | T(58) = | 2.73 |  | 0.008275 | 0.051306 |
| Connection | Visual.Occipital | SensoriMotor.Lateral l | T(58) = | 2.45 |  | 0.017181 | 0.088766 |
| Connection | Visual.Occipital | SensoriMotor.Superior | T(58) = | 2.19 |  | 0.032373 | 0.125447 |
|  |  |  |  |  |  |  |  |
| **Cluster 19** |  |  |  | **6.26** | **0** | **0.006603** |  |
| Connection | Language.IFG l | DefaultMode.PCC | T(58) = | 4.73 |  | 0.000015 | 0.000464 |
| Connection | Language.IFG l | DefaultMode.MPFC | T(58) = | 2.86 |  | 0.005848 | 0.022661 |
| Connection | Language.IFG l | DefaultMode.LP l | T(58) = | 2.52 |  | 0.014658 | 0.040835 |
| Connection | Language.IFG l | DefaultMode.LP r | T(58) = | 2.34 |  | 0.022634 | 0.050119 |
| Connection | Language.pSTG l | DefaultMode.MPFC | T(58) = | 2.03 |  | 0.047269 | 0.104667 |
| Connection | Language.IFG r | DefaultMode.PCC | T(58) = | 2.16 |  | 0.035214 | 0.136454 |
|  |  |  |  |  |  |  |  |
| **Cluster 20** |  |  |  | **6.09** | **0** | **0.00724** |  |
| Connection | Visual.Medial | DorsalAttention.IPS r | T(58) = | 4.82 |  | 0.000011 | 0.000271 |
| Connection | Visual.Medial | DorsalAttention.IPS l | T(58) = | 4.11 |  | 0.000127 | 0.000657 |
| Connection | Visual.Occipital | DorsalAttention.IPS r | T(58) = | 3.92 |  | 0.000239 | 0.003704 |
| Connection | Visual.Occipital | DorsalAttention.IPS l | T(58) = | 3.49 |  | 0.000916 | 0.009461 |
| Connection | Visual.Medial | DorsalAttention.FEF l | T(58) = | 2.97 |  | 0.004273 | 0.013006 |
| Connection | Visual.Medial | DorsalAttention.FEF r | T(58) = | 2.58 |  | 0.012572 | 0.032479 |
| Connection | Visual.Lateral r | DorsalAttention.FEF l | T(58) = | 2.53 |  | 0.014273 | 0.055307 |
| Connection | Visual.Lateral r | DorsalAttention.IPS r | T(58) = | 2.21 |  | 0.031063 | 0.106994 |
| Connection | Visual.Lateral r | DorsalAttention.IPS l | T(58) = | 2.16 |  | 0.034888 | 0.108152 |
| Connection | Visual.Lateral l | DorsalAttention.IPS r | T(58) = | 2.21 |  | 0.031006 | 0.1602 |
| Connection | Visual.Lateral l | DorsalAttention.IPS l | T(58) = | 2.02 |  | 0.047966 | 0.165215 |
|  |  |  |  |  |  |  |  |
| **Cluster 21** |  |  |  | **5.72** | **0.01** | **0.009338** |  |
| Connection | FrontoParietal.LPFC l | DefaultMode.MPFC | T(58) = | 3.41 |  | 0.001198 | 0.009284 |
| Connection | FrontoParietal.PPC r | DefaultMode.LP l | T(58) = | -2.18 |  | 0.033304 | 0.114712 |
|  |  |  |  |  |  |  |  |
| **Cluster 22** |  |  |  | **5.19** | **0.01** | **0.013853** |  |
| Connection | DorsalAttention.IPS r | Cerebellar.Posterior | T(58) = | -3.16 |  | 0.002486 | 0.012846 |
| Connection | DorsalAttention.IPS l | Cerebellar.Posterior | T(58) = | -2.95 |  | 0.004641 | 0.025115 |
| Connection | DorsalAttention.IPS r | Cerebellar.Anterior | T(58) = | -2.16 |  | 0.035106 | 0.072553 |
| Connection | DorsalAttention.IPS l | Cerebellar.Anterior | T(58) = | -2.08 |  | 0.041796 | 0.099638 |
|  |  |  |  |  |  |  |  |
| **Cluster 23** |  |  |  | **4.45** | **0.02** | **0.025097** |  |
| Connection | Visual.Medial | FrontoParietal.LPFC l | T(58) = | 3.88 |  | 0.000269 | 0.001193 |
| Connection | Visual.Medial | FrontoParietal.LPFC r | T(58) = | 2.48 |  | 0.016057 | 0.035556 |
| Connection | Visual.Occipital | FrontoParietal.LPFC l | T(58) = | 2.07 |  | 0.043169 | 0.136055 |
|  |  |  |  |  |  |  |  |
| **Cluster 24** |  |  |  | **4.25** | **0.02** | **0.028464** |  |
| Connection | Salience.SMG r | DefaultMode.LP l | T(58) = | -3.54 |  | 0.000785 | 0.004057 |
| Connection | Salience.Ainsula r | DefaultMode.LP l | T(58) = | -2.4 |  | 0.019609 | 0.04342 |
| Connection | Salience.RPFC r | DefaultMode.MPFC | T(58) = | -2.4 |  | 0.019574 | 0.050566 |
| Connection | Salience.SMG l | DefaultMode.LP r | T(58) = | -2.34 |  | 0.022968 | 0.07911 |
|  |  |  |  |  |  |  |  |
| **Cluster 25** |  |  |  | **3.87** | **0.03** | **0.038217** |  |
| Connection | FrontoParietal.PPC l | FrontoParietal.PPC r | T(58) = | -4.19 |  | 0.000095 | 0.001478 |
| Connection | FrontoParietal.LPFC l | FrontoParietal.PPC r | T(58) = | -3.24 |  | 0.001962 | 0.012161 |
|  |  |  |  |  |  |  |  |
| **Cluster 26** |  |  |  | **3.76** | **0.03** | **0.040666** |  |
| Connection | DorsalAttention.FEF r | FrontoParietal.LPFC r | T(58) = | 3.68 |  | 0.000514 | 0.007961 |
| Connection | DorsalAttention.IPS r | FrontoParietal.LPFC l | T(58) = | -3.07 |  | 0.003287 | 0.013148 |
| Connection | DorsalAttention.FEF l | FrontoParietal.LPFC r | T(58) = | 2.8 |  | 0.006847 | 0.05834 |
| Connection | DorsalAttention.IPS l | FrontoParietal.LPFC l | T(58) = | -2.43 |  | 0.018325 | 0.06312 |
| Connection | DorsalAttention.FEF l | FrontoParietal.LPFC l | T(58) = | 2.19 |  | 0.032249 | 0.099972 |
|  |  |  |  |  |  |  |  |
| **Cluster 27** |  |  |  | **3.53** | **0.04** | **0.047998** |  |
| Connection | SensoriMotor.Lateral r | DefaultMode.PCC | T(58) = | 2.8 |  | 0.006886 | 0.019407 |
| Connection | SensoriMotor.Lateral l | DefaultMode.PCC | T(58) = | 2.78 |  | 0.00723 | 0.021464 |
| Connection | SensoriMotor.Superior | DefaultMode.PCC | T(58) = | 2.14 |  | 0.036392 | 0.063106 |
|  |  |  |  |  |  |  |  |
| p-unc (p value uncorrected)  p-FDR (p value corrected using False Discovery Rate)  p-FWE ( p value corrected using Family Wise Error Rate) | | | | | | | |
