# Supplementary material for: Resting-State Functional Connectivity of the Ageing Female Brain—Differences Between Young and Elderly Female Adults on Multislice Short TR rs-fMRI
Source: Front Neurol. 2021 Jul 12;12:645974. doi: 10.3389/fneur.2021.645974 (PMC8311596; doi:10.3389/fneur.2021.645974)
Supplement: Supplementary file 2 [file Table_2.docx]

**Supplementary Table 2.** Differences between Young Adults vs Old Adults calculated using Network Based Statistics (NBS) method.

| **Network Based Statistics difference YoungAdults vs OldAdults** | | | | | | |
| --- | --- | --- | --- | --- | --- | --- |
|  |  |  |  |  |  |  |
|  |  |  |  | **p-unc** | **p-FDR** | **p-FWE** |
| **NBS Analysis** |  | **Score =** | 291.23 | 0 | 0 | 0 |
|  |  | **Mass =** | 2369.3 | 0 | 0 | 0 |
|  |  | **Size =** | 132 | 0 | 0 | 0 |
| SensoriMotor.Superior | SensoriMotor.Lateral l |  | T(58) = | 5.59 | 0.000001 | 0.000219 |
| FrontoParietal.PPC l | Cerebellar.Anterior |  | T(58) = | 5.49 | 0.000001 | 0.000219 |
| SensoriMotor.Superior | SensoriMotor.Lateral r |  | T(58) = | 5.39 | 0.000001 | 0.000219 |
| Salience.ACC | Language.pSTG r |  | T(58) = | 5.32 | 0.000002 | 0.000219 |
| Cerebellar.Posterior | DefaultMode.LP l |  | T(58) = | 5.23 | 0.000002 | 0.000239 |
| Cerebellar.Anterior | DefaultMode.LP r |  | T(58) = | 5.13 | 0.000004 | 0.00027 |
| Cerebellar.Anterior | DefaultMode.LP l |  | T(58) = | 5.07 | 0.000004 | 0.00027 |
| Salience.SMG r | Salience.ACC |  | T(58) = | 5.07 | 0.000004 | 0.00027 |
| Salience.SMG r | Cerebellar.Posterior |  | T(58) = | -4.95 | 0.000007 | 0.000359 |
| Salience.Ainsula r | Salience.ACC |  | T(58) = | 4.93 | 0.000007 | 0.000359 |
| Salience.SMG r | FrontoParietal.LPFC l |  | T(58) = | -4.9 | 0.000008 | 0.000362 |
| Visual.Medial | DorsalAttention.IPS r |  | T(58) = | 4.82 | 0.000011 | 0.000448 |
| Salience.ACC | Language.pSTG l |  | T(58) = | 4.75 | 0.000014 | 0.000532 |
| Language.IFG l | DefaultMode.PCC |  | T(58) = | 4.73 | 0.000015 | 0.000533 |
| Salience.RPFC r | Language.pSTG r |  | T(58) = | 4.7 | 0.000016 | 0.000541 |
| Visual.Lateral r | Visual.Medial |  | T(58) = | 4.64 | 0.00002 | 0.000624 |
| Language.pSTG l | Cerebellar.Anterior |  | T(58) = | 4.62 | 0.000022 | 0.000626 |
| Cerebellar.Anterior | DefaultMode.PCC |  | T(58) = | 4.61 | 0.000023 | 0.000626 |
| Visual.Medial | SensoriMotor.Lateral r |  | T(58) = | 4.57 | 0.000026 | 0.000684 |
| Visual.Occipital | DefaultMode.PCC |  | T(58) = | 4.53 | 0.00003 | 0.000735 |
| DorsalAttention.IPS r | Language.IFG l |  | T(58) = | -4.48 | 0.000035 | 0.000836 |
| Language.IFG l | Cerebellar.Posterior |  | T(58) = | 4.41 | 0.000045 | 0.000973 |
| Visual.Lateral l | Visual.Medial |  | T(58) = | 4.41 | 0.000045 | 0.000973 |
| Salience.SMG l | Salience.ACC |  | T(58) = | 4.36 | 0.000054 | 0.001123 |
| Cerebellar.Posterior | DefaultMode.LP r |  | T(58) = | 4.34 | 0.000058 | 0.001144 |
| Salience.SMG r | Language.IFG l |  | T(58) = | -4.33 | 0.00006 | 0.001144 |
| Visual.Lateral r | DefaultMode.PCC |  | T(58) = | 4.28 | 0.000071 | 0.001297 |
| Visual.Medial | SensoriMotor.Lateral l |  | T(58) = | 4.22 | 0.000086 | 0.001478 |
| Visual.Lateral l | DefaultMode.PCC |  | T(58) = | 4.2 | 0.000093 | 0.001478 |
| Salience.Ainsula r | Salience.RPFC r |  | T(58) = | 4.2 | 0.000094 | 0.001478 |
| DefaultMode.MPFC | DefaultMode.LP l |  | T(58) = | 4.2 | 0.000095 | 0.001478 |
| FrontoParietal.PPC l | FrontoParietal.PPC r |  | T(58) = | -4.19 | 0.000095 | 0.001478 |
| SensoriMotor.Lateral r | Cerebellar.Anterior |  | T(58) = | -4.14 | 0.000113 | 0.001694 |
| Visual.Medial | DorsalAttention.IPS l |  | T(58) = | 4.11 | 0.000127 | 0.001854 |
| Salience.Ainsula r | Salience.RPFC l |  | T(58) = | 4.07 | 0.000144 | 0.002042 |
| Language.IFG l | Cerebellar.Anterior |  | T(58) = | 4.03 | 0.000164 | 0.002259 |
| Salience.RPFC l | Language.pSTG l |  | T(58) = | 4 | 0.00018 | 0.00242 |
| Salience.Ainsula l | Language.pSTG l |  | T(58) = | 3.92 | 0.000236 | 0.003039 |
| Visual.Occipital | DorsalAttention.IPS r |  | T(58) = | 3.92 | 0.000239 | 0.003039 |
| Visual.Medial | FrontoParietal.LPFC l |  | T(58) = | 3.88 | 0.000269 | 0.003295 |
| Salience.Ainsula r | Language.pSTG r |  | T(58) = | 3.88 | 0.000272 | 0.003295 |
| DorsalAttention.IPS l | SensoriMotor.Superior |  | T(58) = | 3.85 | 0.000293 | 0.00346 |
| DorsalAttention.FEF r | DefaultMode.PCC |  | T(58) = | 3.8 | 0.000351 | 0.004045 |
| SensoriMotor.Lateral r | Salience.RPFC r |  | T(58) = | 3.75 | 0.000413 | 0.004661 |
| SensoriMotor.Lateral r | Salience.ACC |  | T(58) = | 3.73 | 0.000431 | 0.004745 |
| Language.pSTG l | Cerebellar.Posterior |  | T(58) = | 3.73 | 0.000441 | 0.004757 |
| DefaultMode.MPFC | DefaultMode.LP r |  | T(58) = | 3.72 | 0.000451 | 0.004759 |
| Salience.Ainsula r | Cerebellar.Posterior |  | T(58) = | -3.69 | 0.000496 | 0.005019 |
| SensoriMotor.Superior | Salience.SMG r |  | T(58) = | 3.68 | 0.000507 | 0.005019 |
| DorsalAttention.FEF r | FrontoParietal.LPFC r |  | T(58) = | 3.68 | 0.000514 | 0.005019 |
| Salience.SMG l | Language.IFG l |  | T(58) = | -3.68 | 0.000518 | 0.005019 |
| Language.IFG r | Cerebellar.Anterior |  | T(58) = | 3.67 | 0.000526 | 0.005019 |
| Salience.SMG l | FrontoParietal.LPFC l |  | T(58) = | -3.66 | 0.000554 | 0.005187 |
| SensoriMotor.Lateral l | Cerebellar.Anterior |  | T(58) = | -3.58 | 0.000704 | 0.006464 |
| Language.pSTG l | Language.IFG l |  | T(58) = | 3.56 | 0.000746 | 0.00673 |
| Salience.SMG r | DefaultMode.LP l |  | T(58) = | -3.54 | 0.000785 | 0.006954 |
| Salience.SMG l | Cerebellar.Posterior |  | T(58) = | -3.54 | 0.000803 | 0.00699 |
| Salience.RPFC l | Language.pSTG r |  | T(58) = | 3.52 | 0.00086 | 0.007294 |
| SensoriMotor.Lateral r | Salience.RPFC l |  | T(58) = | 3.51 | 0.000876 | 0.007294 |
| DorsalAttention.IPS r | Salience.RPFC r |  | T(58) = | 3.5 | 0.000891 | 0.007294 |
| Visual.Occipital | DorsalAttention.IPS l |  | T(58) = | 3.49 | 0.000916 | 0.007294 |
| Salience.ACC | FrontoParietal.PPC r |  | T(58) = | 3.49 | 0.000916 | 0.007294 |
| Salience.Ainsula l | Salience.RPFC l |  | T(58) = | 3.49 | 0.000938 | 0.007294 |
| Visual.Lateral r | SensoriMotor.Lateral l |  | T(58) = | 3.49 | 0.000941 | 0.007294 |
| Salience.SMG l | Salience.RPFC r |  | T(58) = | 3.48 | 0.000965 | 0.007365 |
| Salience.Ainsula l | Language.pSTG r |  | T(58) = | 3.47 | 0.000998 | 0.007498 |
